# Supplementary material for: Toxic Potential of Cerrado Plants on Different Organisms
Source: Int J Mol Sci. 2022 Mar 22;23(7):3413. doi: 10.3390/ijms23073413 (PMC8998518; doi:10.3390/ijms23073413)
Supplement: Supplementary file 1 [file ijms-23-03413-s001.zip › ijms-1410444-supplementary.pdf]

International Journal of *Molecular Sciences*

Review

Toxic potential of Cerrado plants on different organisms

Jamira Rocha [1], Fernanda Carneiro[2], Amanda Fernandes[3], Jéssyca Morais[1], Leonardo Borges[1], Lee Chen-Chen[3], Luciane Almeida[1], and Elisa Flávia Bailão*[1]

1 Laboratório de Biotecnologia, Câmpus Central, Universidade Estadual de Goiás, CEP:75132-903, Anápolis, GO, Brazil
jamiradias@gmail.com (J.D.R.); jessycamoreiramorais@gmail.com (J.M.M.); leonardo.borges@ueg.br (L.L.B.); luciane.almeida@ueg.br (L.M.d.A.)
2 Laboratório de Ficologia, Unidade Universitária de Goiânia-Laranjeiras, Universidade Estadual de Goiás, CEP: 74863-250, Goiânia, GO, Brazil fermelcar@gmail.com
3 Laboratório de Radiobiologia e Mutagênese, Departamento de Genética, Instituto de Ciências Biológicas I, Universidade Federal de Goiás, CEP: 74045-155, Goiânia, GO, Brazil fer.amanda7@gmail.com (A.S.F.); chenleego@yahoo.com.br (L.C.-C.)

* Correspondence: elisaflavia@gmail.comor elisa.flavia@ueg.br; Tel.: +55-(62)-3328-1151

Abstract: Cerrado has plenty of compounds that have been used as biopesticides, herbicides, cosmetics, medicines, and others due to their highly toxic potential. So, this review aims to bring information about the toxicity of Cerrado plants. For this, a review was performed using PubMed, Science Direct, and Web Of Science databases. After applying exclusion criteria, 187 articles published in the last 20 years were selected and analyzed. Detailed information about extract preparation, part of the plant used, dose/ concentration tested, model system, and assay employed was provided for different toxic activities described in the literature, namely cytotoxic, genotoxic, mutagenic, antibacterial, antifungal, antiviral, insecticidal, antiparasitic, and molluscicidal. In addition, the steps to execute research about plant toxicity and the more common methods employed were discussed. This review synthesized and organized the available research on the toxic effects of Cerrado plants, which could contribute to the future design of new environmental-safe products

Keywords: Brazilian savanna; chemical compounds; microorganisms; natural products; plant extract; tumor cells.

Citation: Rocha, J.D.; Carneiro, F.M.; Fernandes, A.S.; Morais, J.M.; Borges, L.L.; Chen-Chen, L.; de Almeida, L. M.; Bailão, E.F.L.C. Toxic Potential of Cerrado Plants on Different Organisms. *Int. J. Mol. Sci.* **2022**, *23*, 3413. https://doi.org/10.3390/10.3390/ijms23063413

Academic Editor: Chun-Tao Che and Hongjie Zhang

Received: 21 September 2021
Accepted: 27 October 2021
Published: 22 March 2022

Publisher's Note: MDPI stays neutral with regard to jurisdictional claims in published maps and institutional affiliations.

Copyright: © 2021 by the authors. Licensee MDPI, Basel, Switzerland. This article is an open access article distributed under the terms and conditions of the Creative Commons Attribution (CC BY) license (http://creativecommons.org/licenses/by/4.0/).

(1)

(2)

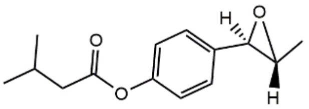

(3)

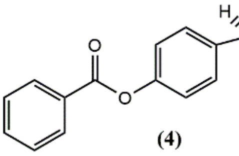

(4)

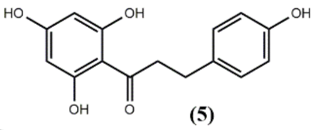

(5)

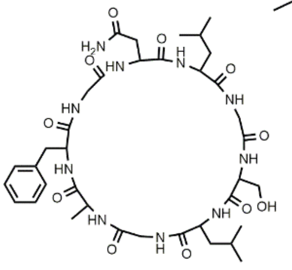

(6)

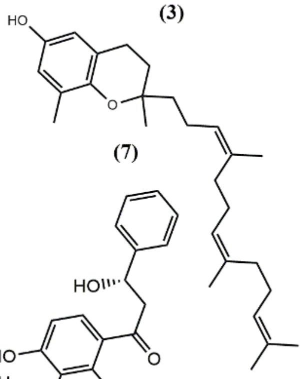

(7)

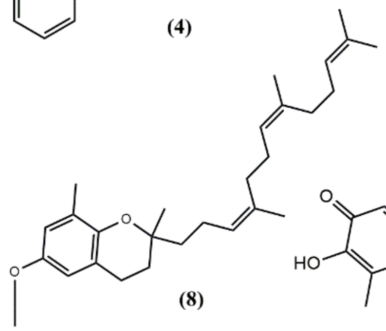

(8)

d-tocotrienol

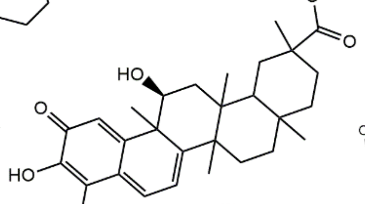

(9)

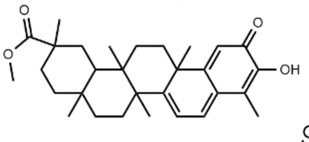

(10)

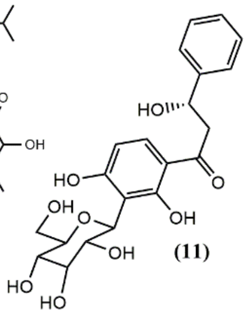

(11)

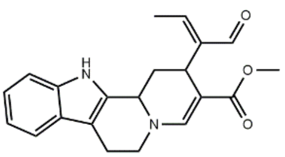

(12)

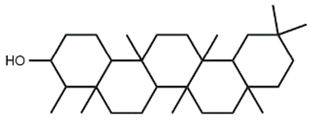

(13)

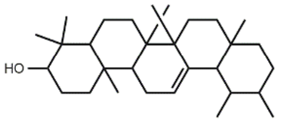

(14)

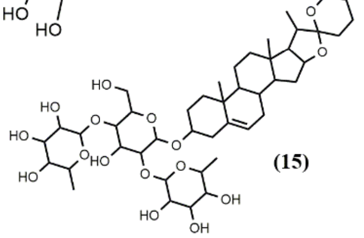

(15)

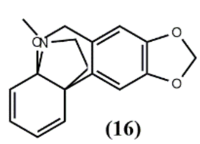

(16)

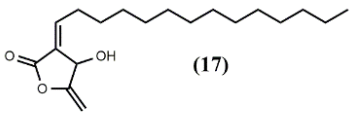

(17)

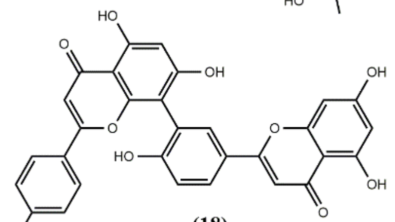

(18)

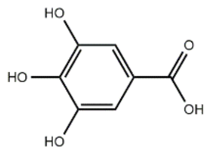

(19)

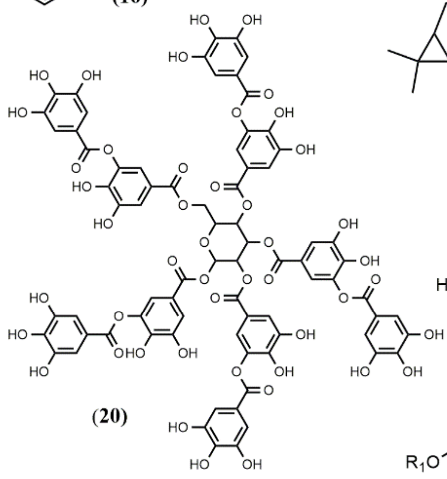

(20)

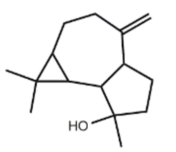

(21)

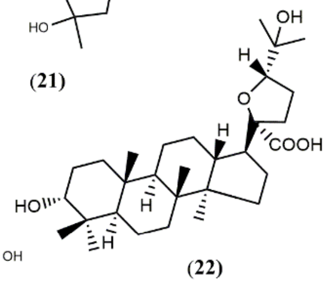

(22)

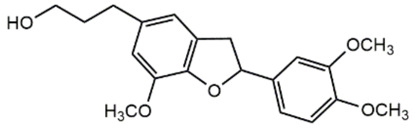

(23)

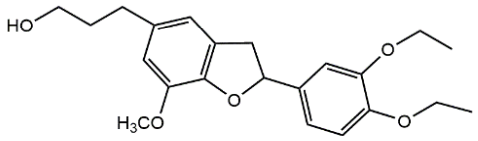

(24)

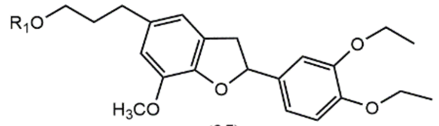

(25)

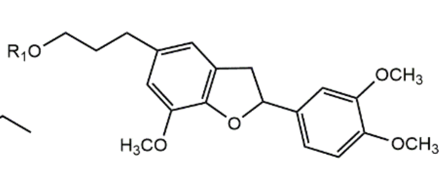

(26)

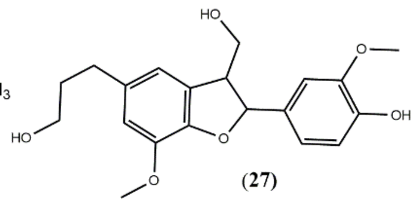

(27)

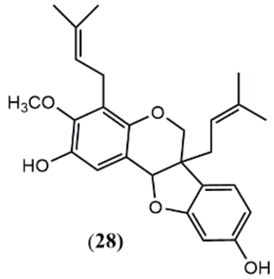

(28)

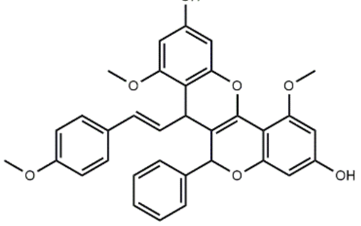

(29)

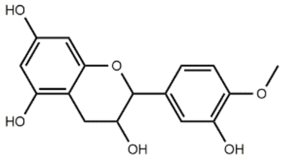

(30)

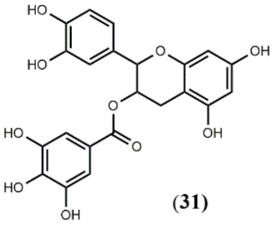

(31)

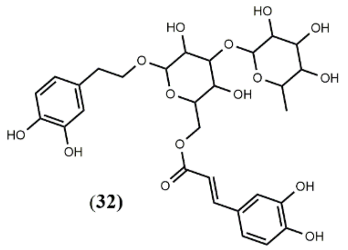

(32)

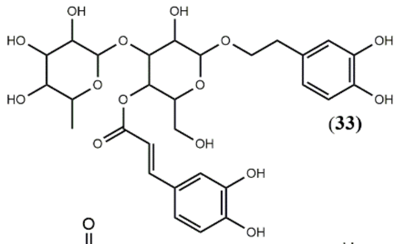

(33)

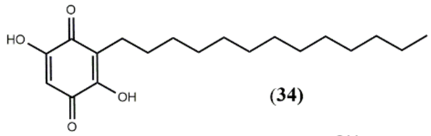

(34)

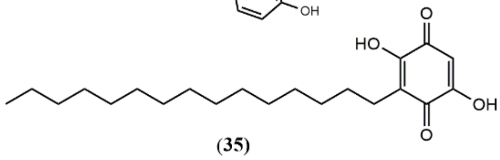

(35)

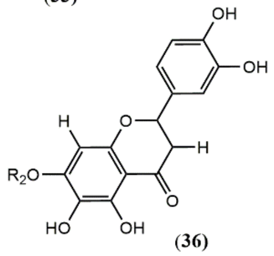

(36)

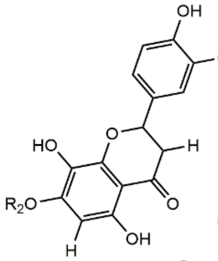

(37)

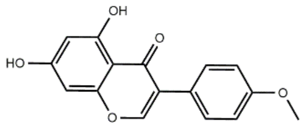

(38)

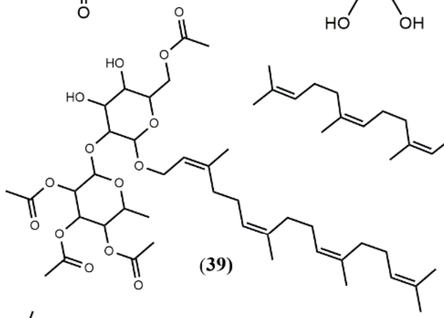

(39)

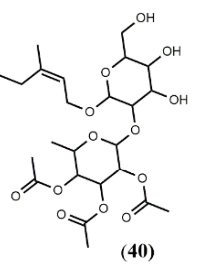

(40)

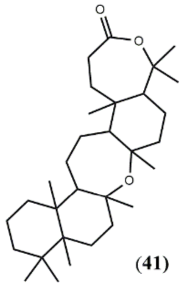

(41)

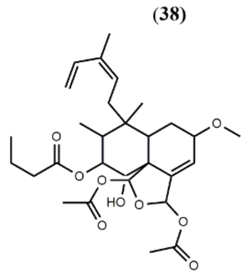

(42)

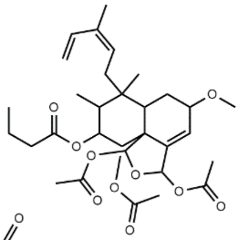

(43)

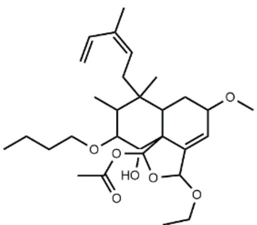

(44)

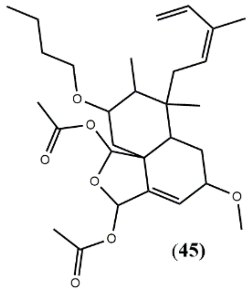

(45)

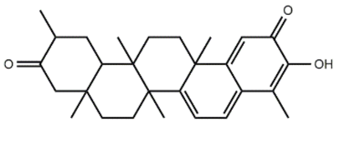

(46)

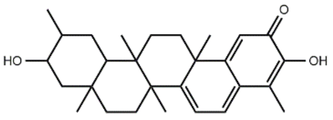

(47)

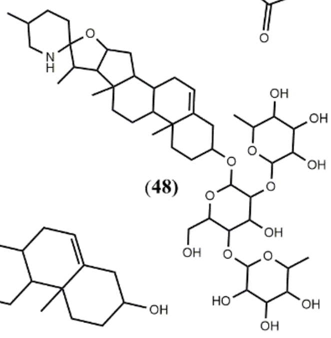

(48)

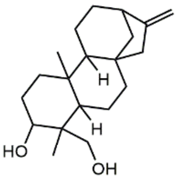

(49)

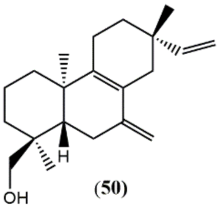

(50)

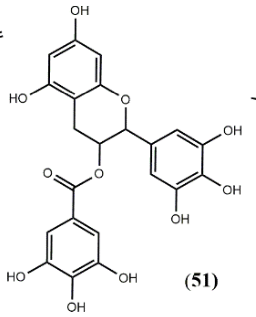

(51)

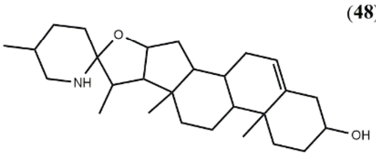

(52)

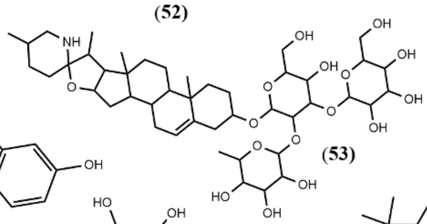

(53)

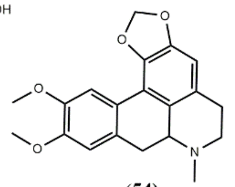

(54)

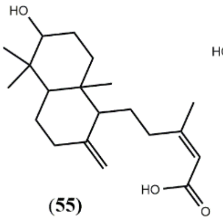

(55)

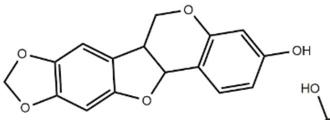

(56)

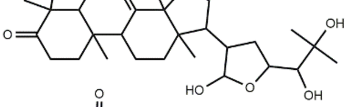

(57)

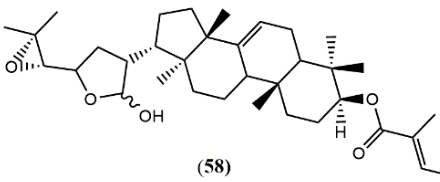

(58)

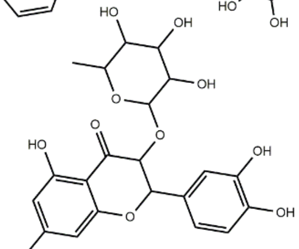

(59)

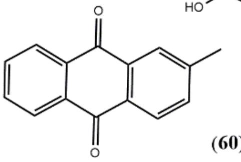

(60)

Figure S1. Molecular structure of secondary metabolites isolated from Cerrado plant species. For names refers to Table S10. -R1=glucose; -R2=glucopyranosyl.

Table S1 Cytotoxic activity from different species of Cerrado. Information about part from plant and extract preparation, dose tested, model and method analyzed.

Family/Species	Part[1]	Extract[2]	Dose/Concentration	Activity against	Technique	Model[3]	Reference
**Annonaceae**							
Annona crassiflora Mart.	RB, RW or F	A, Eth or MA	5.36-230.12 and 6.0-24.9 µg/mL	Tumor cells	SRB and MTT assay	HCT-8, MDA-MB-435, SF-295, U251, MCF-7NCIADR/RES, NCI-H460, PC-3, OVCAR-03, HT-29, K562, and HaCaT	[1,2]
**Asteraceae**							
Baccharis dracunculifolia DC.	L	EA	200.0 and 400.0 $\mu g/mL^{-1}$	Murine cells	Trypan Blue Assay	V79 cells	[3]
**Anacardiaceae**							
Schinus terebinthifolius var. raddianus Engl.	L	HE or DM	1.1-41.8 µg/mL	Tumor cells	MTT assay	HCT-8, MDA-MB-435, and SF-295	[1]
**Annonaceae**							
Xylopia aromatica (Lam.) Mart.	RW	DM	5.9-19.1 µg/mL	Tumor cells	MTT assay	HCT-8, MDA-MB-435, SF-295, and HL60	[1]
**Apocynaceae**							
Himatanthus drasticus (Mart.) Plumel	La	Aq	50 and 100 µg/mL	Tumor cells	MTT assay	S-180 cells	[4]
**Asteraceae**							
Vernonanthura polyanthes (Sprengel) Vega & Dematteis	L	Aq, n-BF or EA	20-40 and 0.25, 0.5, and 1 mg/mL	Crustacean, onion and human cells	*Artemia salina, Allium cepa* and, Trypan Blue assay	*Artemia salina*, Meristematic cells and Human lymphocytes	[5,6]
Eremanthus incanus (Less.) Less.	AE	EA or Eth	50 µg/mL	Tumor cells	MTT assay	HCT 116, OVCAR 8 and, SF-295	[7]

**Bignoniaceae**							
Distictella elongata (Vahl) Urb.	RO	HE	100 µg/mL^{-1}	Tumor cells	Neutral Red	SK-ME and SK-OV-3	[8]
Pyrostegia venusta (Ker Gawl.) Miers	FL	Hep	28.96 mg/mL or 41.08 µg/mL	Tumor cells	MTT assay	B16F10-Nex2	[9]
**Bixaceae**							
Cochlospermum regium (Mart. ex Schrank) Pilg.	R	Aq	19, 38, 76 and 114 mg/kg	Murine cells	Micronucleus test	Erythrocytes	[10]
**Calophyllaceae**							
Kielmeyera coriacea Mart. & Zucc.	L, SB, SW or R	Chl or HE	5-40 µg/mL	Tumor and murine cells	MTT and Trypan Blue assay	SKMel 28 or A2058 cells and B16F10-Nex2, HCT-8, MDA-MB-435, SF-295, HL-60, and SF-295 cells lines	[1,11,12]
Calophyllum brasiliense Cambess.	R	DM	3.0- 17.6 µg/mL	Tumor cells	MTT assay	Cell lines: HCT-8, MDA-MB-435, and SF-295	[1]
**Caryocaraceae**							
Caryocar brasiliense Cambess.	F	EO	173.7 – 442.7 µg/mL	Tumor cells	MTT assay	4T1 and NIH/3T3	[13]
**Celastraceae**							
Salacia crassifolia (Mart. ex Schult.) G.Don	RW or B	HE	0.3-8.7 µM and 50-150 mg/kg	Tumor and murine cells	Formazan Endpoint assay and Micronucleus test	KM12, A498, HCT-15, UO-31, T-47D, A549, 786-O, NCI-60 and murine erythrocytes	[14,15]
Cheiloclinium cognatum (Miers) A.C.Sm.	Bra		9.92- 55.65 µmol/L-1	Tumor and non-tumor cells	MTT assay	ATCC TIB-202 (THP-1), ATCC CRL-3343 (K562) and PBMCs	[16]

**Clusiaceae**							
Garcinia gardneriana (Planch. & Triana) Zappi	L or F	Eth, HE, Chl, HM or AE	0.25, 2.5, 25 and 250 µg/mL	Tumor and non-tumor cells	SRB assay	B16-F10, MCF-7, 786-0, HT-29 and NIH-3T3	[17]
**Costaceae**							
Costus spiralis (Jacq.) Roscoe	L or S	Aq	18 and 9 mg/mL	Onion cells	*Allium cepa* test	Meristematic cells	[18]
Chamaecostus subsessilis (Nees & Mart.) C.D.Specht & D.W.Stev.	Rh	Chl or Eth	4.6 and 15.1 µg/mL	Tumor cells	MTT assay	HL60	[19]
**Erythroxylaceae**							
Erythroxylum daphnites Mart.	L	Aq, Eth or HE	448.9 and 500 µg/mL	Tumor cells	MTT assay	SCC-9, HaCat and FaDu	[20,21]
Erythroxylum suberosum A.St.-Hil.	L	Aq, Eth or HE	500 µg/mL	Tumor cells	MTT assay	SCC-25, SCC-9, FaDu and, HaCat	
Erythroxylum subrotundum A.St.-Hil.	L	Aq, Eth or HE	500 µg/mL	Tumor cells	MTT assay	SCC-SCC-9	[20]
**Euphorbiaceae**							
Croton velutinus Baill.	R	HE	6.8-19.4 µM/mL	Tumor cells	Alamar Blue assay	MCF7 and HepG2	[22]
Croton urucurana Baill.	La	EA	3.93-5.42 $\mu g/mL^{-1}$ and 50-200 µg/mL	Tumor cells	SRB assay and WST-1 assay	786-0, NCI-ADR/RES and Huh-7	[23,24]
**Fabaceae**							
Eriosema crinitum (Kunth) G.Don	R	DM or Eth	100 µg/mL	Human cells	Trypan blue assay	T cells	[25]
Stryphnodendron adstringens (Mart.) Coville	B	Aq or Eth	65-100 µg/mL and 0.5 mg/plate	Tumor and Bacterial cells	MTT assay and Ames test	PBMC, B16F10-Nex2 and *Salmonella typhimurium* (TA100, TA98, TA97a and, TA102)	[26,27]
Dipteryx alata Vogel	N	C or HD	0.781 and 50 mg/mL	Tumor cells	MTT assay and PrestoBlue assay	HT29	[28]

Hymenaea stigonocarpa Mart. ex Hayne	B	Aq or HD	0.5-1.5 mg/mL and 0.082-0.328g/mL	Onion cells	*Allium cepa* test	Meristematic cells	[29,30]
**Lauraceae**							
Nectandra amazonum Nees	L	EO	58.0 and 29.40 µg/mL	Murine cells	SRB assay	J774.A1 and NIH/3T3	[31]
Nectandra gardneri Meisn.	SB	EO	51.60 and 29.90 µg/mL	Murine cells	SRB assay	J774.A1 and NIH/3T3	[31]
Nectandra hihua (Ruiz & Pav.) Rohwer	L	EO	54.90 and 29.80 µg/mL	Murine cells	SRB assay	J774.A1 and NIH/3T3	[31]
Nectandra lanceolata Nees	L or B	EO	14.6, 67.5, 72.4, 107.0 and 195.2 µg/mL $^{-1}$	Tumor cells	SRB assay	786-0, HT-29 and K562	[32]
Nectandra megapotamica (Spreng.) Mez	L or SB	EO	71- 415.60 µg/mL	Tumor and murine cells	MTT and SRB assays	Human erythrocytes,Vero cells, J774.A1) and NIH/3T3	[31,33]
**Loganiaceae**							
Strychnos pseudoquina A.St.-Hil.	L	M	26.60 mg/plate	Bacterial cells	Ames test	*Salmonella typhimurium* (TA98)	[34]
**Lythraceae**							
Lafoensia pacari A.St.-Hil.	B	M, Mac, or Dec	18.32- 21.08 µg/mL and 0.1-1000 mg/mL	Tumor cells	MTT, Clonogenic and Trypan blue assays	HRT-18, SH-SY5Y, HeLa, HRT-18, U-937, Daudi, T-cell and, Jurkat	[35,36]
**Myrtaceae**							
Eugenia dysenterica (Mart.) DC.	L	Aq or Eth	15.6 µg/mL and 100, 150 and 200 mg/kg	Tumor and murine cells	XTT assay and Micronucleus test	SH-SY5Y and Murine erythrocytes	[37,38]
Eugenia uniflora L.	L	EO	76.5-102,2 µg/mL	Tumor and non-tumor cells	XTT assay	MO59 J, HeLa, MCF-7 and GM07492 A	[39]
Myrcia bella Cambess.	L	HA	200- 500 mg/mL	Human cells	MTT assay	ACP02	[40]
Psidium brownianum Mart. ex DC.	L	Aq	1000 µg/mL	Murine cells	Resazurin Assay	NCTC-929	[41]

**Salicaceae**							
Casearia sylvestris Sw.	L, SB, RB, RD, F or SW	Eth or HE	0.1- 33.7 and 111.3-149.3 µg/mL	Tumor and murine cells	MTT assay	HCT-8, MDA-MB-435, SF-295 and J774	[1,42]
**Sapindaceae**							
Cupania vernalis Cambess.	L, S, B, SB or RB	Eth or HE	4.0- 4.2 and 66.5-160. 0 µg/mL	Tumor and murine cells	MTT assay	HCT-8, MDA-MB-435, SF-295 and J774	[1,42]
Serjania lethalis A.St.-Hil.	L, S, B, SB or RB	Eth or HE	8.0- 205.1 µg/mL	Tumor and Murine cells	MTT assay	J774, HCT-8, MDA-MB-435, and SF-295	[1,42]
**Sapotaceae**							
Pouteria torta (Mart.) Radlk.	L	Aq, Eth or HE	125 and 500 µg/mL	Tumor cells	MTT assay and Violet crystal test	FaDu, OSCC-3 or MCF-7	[20,43]
Pouteria ramiflora (Mart.) Radlk.	L	Aq, Eth or HE	500 µg/mL	Tumor cells	MTT assay	FaDu and HaCat	[20]
**Simaroubaceae**							
Simarouba versicolor A.St.-Hil.	RB or F	Eth or HE	0.2- 16.6 µg/mL	Tumor cells	MTT assay	HCT-8, MDA-MB-435 and, SF-295	[1]
**Solanaceae**							
Solanum lycocarpum A.St.-Hil.	F	Eth	5, 10, 25, 50 and 80 mg/kg	Murine cells	Micronucleus test	Murine erythrocytes	[38]
**Verbenaceae**							
Lippia salviaefolia Cham.	L or S	Eth	20-40 µM	Tumor cells	MTT assay	HEK-293 and M14	[44]

Part used[1](AP= aerial parts, Bra= Branches, F= Fruits, FL= Flowers, La= Latex, N= Nuts, L= Leaves, RB=root bark; R= Root; Root Wood= RW; Rh=Rhizome, and S= Stem; Stem Bark= SB; Bark= B).
Type of extract[2] (Aqueous= Aq; Dichloromethane= DM; Ethanolic= Eth; Ethyl-acetate= EA; Methanol-acetate= MA; Hexane= HE; n-butanol= n-BF; Heptane= Hep; Chloroform= Chl; Essential oils= EO; Hydromethanolic=HM; Crude= C; Hydrolyzed= HD; Methanol= M; Macerated= Mac, Decoction= Dec; Hydroalcoholic= HÁ; Hydroethanolic= HEth).

Cells lines[3]: MDA-MB-435 (melanoma), HCT-8 (colon), SF-295 (glioblastoma), NCI-ADR/RES (multidrug resistant ovary adenocarcinoma), U251 (glioma), MCF-7 (breast), NCI-H460 (non-small cell lung cancer), PC-3 (prostate) , OVCAR-03 (ovary), HT-29 (colon), K562 (leukemia), HaCaT (spontaneously transformed kerati- nocytes from histologically normal skin), V79 cells (Chinese hamster lung fi broblasts), S-180 (Sarcoma-180), HCT-116 and OVCAR-8 (human cancer cell lines), SK-MEL (melanoma), SK-OV-3 (ovary adenocarcinoma), B16F10-Nex2 (murine melanoma), 471 (breast), NIH/3T3 (non-tumoral), Colo205 and KM12 (colon cancer), A498 and U031 (renal cancer), HEP3B and SKHEP (liver cancer) MG63 and MG63.3 (osteosarcoma), K562 and THP-1 (leukemia), B16-F10 (Murine melanoma), 786-0 (kidney adenocarcinoma), SCC-25 and SCC-9 (Oral cavity squamous cell carcinoma), FaDu (carcinoma) HaCat (keratinocyte), Huh-7 (human hepatocarcinoma), PBMC (Peripheral blood mononuclear cells), Murine macrophage (J774.A1), K562 (leukemia), Jurkat, and Daudi (leukemic cell lines), SH-SY5Y (neuroblastoma), MO59J (human glioblastoma), GM07492 A (lung fibroblasts), HeLa (human cervical adenocarcinoma), HL-60 (leukemia), HepG2 (hepatocyte carcinoma), M14 (melanoma) and, NCI-60 (Mammalian Cell Lines),

Table S2 Mutagenic and genotoxic activities from different species of Cerrado. Information about part of plant and extract preparation, dose tested, model and method analyzed.

Family/Species	Part¹	Extract²	Dose/Concentration	Activity against	Technique	Model	Reference
**Apocynaceae**							
Himatanthus drasticus (Mart.) Plumel	L	Aq	2000 mg/kg	Murine cells	Assay comet	Erythrocytes	[4]
**Asteraceae**							
Baccharis dracunculifolia DC.	L	EA	50 and 100 µg/mL	Murine and Mammalian cells	Assay comet and Chromosomal aberration assay	V79 and CHO9 cells	[3,45]
Vernonanthura polyanthes (Sprengel) Vega & Dematteis	L	Aq, n-BF or EA	0.25- 1 mg/mL	Human cells	CometChip	Human lymphocytes	[6]
**Bignoniaceae**							
Arrabidaea brachypoda (DC.) Bureau	L, S or R	Aq or HA	12-24 mg/plate	Bacterial cells	Ames test	*Salmonella typhimurium* (TA98)	[46]
**Bixaceae**							
Cochlospermum regium (Mart. ex Schrank) Pilg.	R	Aq	19- 114 mg.kg	Murine cells	Micronucleus test	Erythrocytes	[10]
**Combretaceae**							
Terminalia argentea Mart. & Zucc.	L	HEth	10-100 µg/mL	Tumor cells	Assay comet and Micronucleus test	CHO-K1 cells	[47]
**Dilleniaceae**							
Davilla elliptica A.St.-Hil.	L	Aq, M, HA or EA	0-84 mg/plate	Bacterial cells	Ames test	*Salmonella typhimurium* (TA98 and TA97a)	[48]
**Dilleniaceae**							
Davilla nitida (Vahl) Kubitzki	L	Aq, M, HA or EA	0-84 mg/plate	Bacterial cells	Ames test	*Salmonella typhimurium* (TA98 and TA97a)	[49]
**Loganiaceae**							

Strychnos pseudoquina A.St.-Hil.	L	M	3.33-26.60 mg/plate and 1800 mg/kg	Bacterial and human cells	Ames and Micronucleus test	*Salmonella typhimurium* (TA98 and TA100) human reticulocytes	[34]
**Malpighiaceae**							
Byrsonima crassa Nied.	L	M or EA	0-35.5 mg/plate	Bacterial cells	Ames test	*Salmonella typhimurium* (TA98 and TA100)	[50]
**Melastomataceae**							
Mouriri elliptica Mart.	L	M	2.3 – 18.6 mg/plate	Bacterial and Murine cells	Ames and Micronucleus test	*Salmonella typhimurium* (TA98, TA100 and TA97a)	[51]
Mouriri pusa Gardner	L	M	2.84-22.70 mg/plate	Bacterial and Murine cells	Ames and Micronucleus test	*Salmonella typhimurium* (TA98, TA100 and TA97a)	[51]
**Myrtaceae**							
Eugenia dysenterica (Mart.) DC.	L	Eth	150 and 200 mg/kg	Murine cells	Micronucleus test	Erythrocytes	[38]
**Smilacaceae**							
Smilax brasiliensis Spreng.	L	M, DM or EA	250-1000 μg/mL	Onion cells	*Allium cepa* test	Meristematic cells	[52]
**Vochysiaceae**							
Qualea grandiflora Mart.	B	Aq, M or Chl	1.92-15.40 mg/plate	Bacterial and Murine cells	Ames and Micronucleus test	*Salmonella typhimurium* (TA98, TA100, TA97a and TA102)	[48]
Qualea multiflora Mart.	B	Aq, M or Chl	2.07-16.60 mg/plate	Bacterial and Murine cells	Ames and Micronucleus test	*Salmonella typhimurium* (TA98, TA100, TA97a and TA102)	[48]

Part used[1] (AP= aerial parts, Bra= Branches, F= Fruits, FL= Flowers, La= Latex, N= Nuts, L= Leaves, RB=root bark; R= Root; Root Wood= RW; Rh=Rhizome, and S= Stem ; Stem Bark= SB; Bark= B). Type of extract[2] (Aqueous= Aq; Dichloromethane= DM; Ethanolic= Eth; Ethyl-acetate= EA; Methanol-acetate= MA; Hexane= HE; n-butanol= n-BF; Heptane= Hep; Chloroform= Chl; Essential oils=EO; Hydromethanolic=HM; Crude= C; Hydrolyzed= HD; Methanol= M; Macerated= Mac, Decoction= Dec; Hydroalcoholic= HÁ; Hydroethanolic= HEth; Exsudate=EX).

Table S3 Acute and chronic toxicity on murine model. Information about part of plant and extract preparation, dose tested, model and method analyzed.

Family/Species	Part[1]	Extract[2]	Dose/Concentration	Activity against	Technique	Model	Reference
**Asteraceae**							
Lychnophora pinaster Mart.	AP	Eth	125-500 mg/kg	Mice	One single dose by oral route	Swiss	[53]
Lychnophora trichocarpha (Spreng.) Spreng.	AP	Eth	0.5 - 1.5 g/kg	Mice	one single dose by intraperitoneal route	Albino Swiss	[54]
**Fabaceae**							
Dimorphandra mollis Benth	F	HA	2000 mg/kg	Rats	Oral administration for 180 days	Male Wistar	[55]
**Moraceae**							
Brosimum gaudichaudii Trécul	RB	EX	LD50 3517 and 2871.76 mg/kg	Mice	One single dose by oral or intraperitoneal routes	Albino male mice	[56]
**Myrtaceae**							
Campomanesia velutina (Cambess) O. Berg	L or Bra	Aq	300-1200 mg/kg	Mice	One single dose or 14 consecutive days by oral route	Albino Swiss	[57]
**Sapindaceae**							
Serjania marginata Casar.	L	Aq	30-750 mg/kg	Rats	28 consecutive days by oral route	Wistar	[58]

Part used[1] (AP= aerial parts; Bra= Branches; F= Fruits; RB=root bark; R= root; root wood= RW).
Type of extract[2] (Aqueous= Aq; Ethanolic= Eth; Hydroalcoholic= HA; Exsudate=EX).

Table S4. Antibacterial activity from different species of Cerrado. Information about part from plant and extract preparation, dose tested, model and method analyzed

Family/Species	Part[1]	Extract[2]	Dose /Concentration	Activity against	Technique	Experimental model	Reference
**Anacardiaceae**							
Myracrodruon urundeuva M. Allemão	L	HEth	MIC = 2.5 mg/mL	*Streptococcus mutans*	Broth Microdilution Assay	*S. mutans* (ATCC 21175)	[59]
Schinopsis brasiliensis Engl.	L	Eth	MIC = 0.17 mg/mL	*Escherichia coli*	Broth Microdilution Assay	*E. coli* isolates (E2 and E3)	[60]
**Annonaceae**							
Annona coriacea Mart.	L	AC and C	MIC = 3.12--6.25-50 and 100-200 µg/mL	*Streptococcus mutans, Streptococcus mitis , Streptococcus sanguinis* and *Streptococcus salivarius*	Broth Microdilution Assay	*S. mutans* (*ATCC* 25175), *S. mitis* (ATCC 49456), *S. sanguinis* (ATCC 10556) and *S. salivarius* (ATCC 25975)	[61]
Cardiopetalum calophyllum Schltdl.	L	EO	MIC = 100 and 400 µg/mL	*Streptococcus mutans, Streptococcus sanguinis, Streptococcus sobrinus, Bacteroides fragilis* and *Streptococcus mitis*	Microdilution Assay	*S. mutans, S. sanguinis, S. sobrinus, B. fragilis* and *S.mitis*	[62]
**Apocynaceae**							
Hancornia speciosa Gomes	B	Eth	MIC = 50 and 125 µg/mL	*Helicobacter pylori* and *Staphylococcus aureus*	Broth Microdilution Assay	*H. pylori* (ATCC 43504), and *S. aureus* (ATCC 25923)	[63]
Secondatia floribunda A.DC.	SIB	Eth	MIC = 64 and 128 µg/mL	*Staphylococcus aureus and Escherichia coli*	Broth Microdilution Assay	*S. aureus* (ATCC 12692) and *E. coli* (ATCC 25922)	[64]
**Arecaceae**							

Attalea speciosa Mart. ex Spreng.	F or L	FO or EO	MIC = 32-400 µg/mL	*Escherichia coli, Staphylococcus aureus, Streptococcus sanguinis* and *Streptococcus mitis , Streptococcus mutans Agregatibacter actinomycetemcomitans , Actinomyces naeslundii , Porphyromonas gingivalis and Fusobacterium nucleatum.*	Broth Microdilution Assay	*E. coli* Ec 27 (multiresistant); *S. aureus* 358 (multiresistant); *S. sanguinis* (ATCC 10556); *S. mitis* (ATCC 49456), *S. mutans* (ATCC 25175), *A. actinomycetemcomitans* (ATCC 43717), *A. naeslundii* (ATCC 19039), *P. gingivalis* (ATCC 33277) and *F. nucleatum* (ATCC 25586)	[65,66]
Mauritia flexuosa L.f.	S	DM	MIC = 31.3 µg/mL	*Methicillin-susceptible Staphylococcus aureus* and *methicillin-resistant Staphylococcus aureus*	Broth Microdilution Assay	methicillin-susceptible *S. aureus* (MSSA- ATCC 29213) and methicillin-resistant *S. aureus* (MRSA - clinical sample 155)	[67]
**Aristolochiaceae**							
Aristolochia cymbifera Mart. & Zucc.	S, W and R	Eth and HE	MIC = 0.5-4.0 and 125-500 µg/mL	*P. intermedia, P. gingivalis , F.nucleatum, S.mutans, L. casei and S. aureus, S. epidermidis , S. haemolyticus, P. aeruginosa*	Broth Microdilution Assay	*P. intermedia (*ATCC 25611), *P. gingivalis* (ATCC 49417), *F. nucleatum* (ATCC 25586), *S. mutans* (ATCC 25175), *L. casei* (ATCC 4646), *S. aureus*, *S. epidermidis* (10 multi-resistant strains), *S. haemolyticus* (12 multi-resistant), *P. aeruginosa* (21 multi-resistant strains)	[68,69]
**Asteraceae**							
Chromolaena squalida (DC.) R.M.King & H.Rob.	L	EO	MIC = 7.80 µg/mL	*multi–resistant Staphylococcus sp. 841*	Broth Microdilution Assay	*multi–resistant Staphylococcus sp. 841*	[70]
**Bixaceae**							
Cochlospermum regium (Mart. ex Schrank) Pilg.	X, R and L	HEth, EA, Aq and Eth	MIC = 31-500 µg/mL and 1 mg/mL	*Helicobacter pylori, Pseudomonas aeruginosa, Acinetobacter baumannii, Staphylococcus epidermidis, Staphylococcus aureus, Streptococcus agalactiae; Escherichia coli, Klebsiella pneumoniae,* and *Acinetobacter baumannii*	Broth Microdilution Assay	*H. pylori* (ATCC 43504*), P. aeruginosa* (25619), *A. baumannii* (19606), *S. epidermidis* (12228), *S. aureus* (29213), *E. coli* (25922), *S. agalactiae* (13813), *K. pneumoniae* (43816), *P. aeruginosa* (25619), *A. baumannii* (19606)	[71–74]
**Calophyllaceae**							

Kielmeyera lathrophyton Saddi	B	BC	MIC = 250-500 µg/mL	*Staphylococcus aureus* and *Bacillus subtilis*	Microdilution Assay	*S. aureus* (ATCC 25923), *B. subtilis* (ATCC 6623),	[75]
Caryocar brasiliense Cambess.	L	Eth and HEth	MIC = 0.27, 22.50 and 11.25 mg/mL	*Staphylococcus aureus* and *Staphylococcus haemolyticus; Escherichia coli* and *Pseudomonas aeruginosa*	Broth Microdilution and agar diluition Assay	*Staphylococcus* isolates (S178, S135 and S182)	[76,77]
**Dilleniaceae**							
Curatella americana L.	B	BC	MIC = 500 µg/mL	Staphylococcus aureus	Microdilution Assay	*S. aureus* (ATCC 25923)	[75]
Davilla nitida (Vahl) Kubitzki	L	M	MIC = 125 µg/mL	*Helicobacter pylori*	Broth Microdilution Assay	*H. pylori* (ATCC 43504)	[78]
Davilla elliptica A.St.-Hil.	L	M	MIC = 250 µg/mL	*Helicobacter pylori*	Broth Microdilution Assay	*H. pylori*(ATCC 43504)	[78]
**Erythroxylaceae**							
Erythroxylum suberosum A.St.-Hil.	SB	Eth	MIC = 250 µg/mL	*Staphylococcus aureus*	Broth Microdilution Assay	*S. aureus* (ATCC 25923)	[79]
**Euphorbiaceae**							
Alchornea triplinervia (Spreng.) Müll.Arg.	L	M	MIC= 0.25 mg/mL	*Helicobacter pylori*	Broth Microdilution Assay	*H. pylori* (ATCC 43504)	[80]
Croton heliotropiifolius Kunth	L and S	EO	MIC = 62.5 and 500 µg/mL	*Staphylococcus aureus* and *Bacillus subtilis*	Broth Microdilution Assay	*S. aureus* and *B. subtilis*	[81]
**Fabaceae**							
Plathymenia reticulata Benth.	L	M	MIC = 5 mg	*Escherichia coli* and *Pseudomonas aeruginosa*	Agar Diffusion assays	*E. coli* and *P. aeruginosa*	[82]
Stryphnodendron adstringens (Mart.) Coville	L and B	D, M and W	MIC = 0.19-12.5 µg/mL and 5 mg	*Staphylococcus aureus, Pseudomonas aeruginosa, Klebsiella pneumoniae,* and *Escherichia coli*	Broth Microdilution and Agar Diffusion Assay	*S. aureus* (ATCC 25923), *P. aeruginosa* (ATCC 27853), *K. pneumoniae* (ATCC 4352) and *E. coli* (ATCC 25922)	[82,83]
**Lamiaceae**							

Hyptis crenata Pohl ex Benth.	WP	DM	MIC = 62.5 µg/mL	*Staphylococcus aureus* and *Enterococcus faecalis*	Broth Microdilution Assay	*S. aureus* (ATCC 25923) and *E. faecalis* (ATCC 29218)	[79]
Hyptis multibracteata Benth.	AP	HE, EA and HE	MIC = 37.55 and 12.13-23.6 µg/mL	*Pseudomonas aeruginosa* and *Bacillus subtilis*	Broth Microdilution Assay	*P. aeruginosa* (ATCC 10.145) and *B. subtilis* (PY79)	[84]
Hyptis passerina Mart. ex Benth.	L	EO	MIC = 62.5-125 µg/mL	*Staphylococcus aureus* and *Pseudomonas aeruginosa*	Broth Microdilution Assay	*S. aureus* (ATCC 25923) and *P. aeruginosa* (ATCC 15422)	[85]
Hyptis radicans (Pohl) Harley & J.F.B.Pastore	AP	EA	MIC = 140.91 and 195.32 µg/mL	*Bacillus subtilis* and *Pseudomonas aeruginosa*	Broth Microdilution Assay	*B. subtilis* (PY79) and *P. aeruginosa* (ATCC 10.145)	[84]
**Loganiaceae**							
Strychnos pseudoquina A.St.-Hil.	L	EAF	MIC = 75 µg/mL	*Helicobacter pylori*	Broth Microdilution Assay	*H. pylori* (ATCC 43504)	[86]
**Malpighiaceae**							
Byrsonima intermedia A.Juss.	L	M	MIC= 0.125-0.500 mg/mL	*Helicobacter pylori; Staphylococcus aureus and Escherichia coli*	Microdilution Assay	*H. pylori* (ATCC 43504), *S. aureus* (ATCC 25923) and *E. coli* (ATCC 25922)	[87]
**Melastomataceae**							
Miconia albicans (SW.) Triana	L	M	MIC = 5 mg	*Escherichia coli* and *Pseudomonas aeruginosa*	Agar Diffusion Assay	*E. coli* and *P. aeruginosa*	[82]
Mouriri elliptica Mart.	L	M	MIC = 0.025 mg/mL	*Helicobacter pylori*	Broth Microdilution Assay	*H. pylori* (ATCC 43504)	[88]
**Myrtaceae**							
Eugenia dysenterica (Mart.) DC.	P	Pw	MIC = 0.16 mg/mL	*Staphylococcus aureus* and *Listeria monocytogenes*	Broth Microdilution Assay	*S. aureus and L. monocytogenes*	[89]
Eugenia involucrata DC.	L	OE or DM	MIC = 50, 100 and 200 µg/mL	*Prevotella nigrescens, Porphyromonas gingivalis, Streptococcus mutans, Streptococcus mitis, Streptococcus sanguinis, Streptococcus sobrinus* and *Bacteroides fragilis*	Broth Microdilution Assay	*P. nigrescens* (ATCC 33563) and *P. gingivalis* (ATCC 33277), *S. mutans* (ATCC 25175), *S. mitis* (ATCC 49456), *S. sanguinis* (ATCC 10556), *S. sobrinus* (ATCC 33478) and *B. fragilis* (ATCC 25285)	[90]

Eugenia klotzschiana O.Berg	L and FL	EO	MIC = 50 µg/mL	*Streptococcus mutans* and *Prevotella nigrescens*	Broth Microdilution Assay	*S. mutans* (ATCC 25175) and *P. nigrescens* (ATCC 33563)	[91]
Eugenia uniflora L	L	EO	MIC = 100, 200 and 400 µg/mL	*Streptococcus mutans, Stre ptococcus sobrinus, Prevotella nigrescens, Porphyromonas gingivalis, Streptococcus sanguinis* and *Streptococcus mitis*	Broth Microdilution Assay	*S. mutans, S. sobrinus, P. nigrescens, P. gingivalis; S.* and *sanguinis; S. mitis*	[92]
Myrcia bella Cambess	L	HEth	MIC = 300 µg/mL	*Escherichia coli*	Broth Microdilution Assay	*E. coli* (ATCC 11775)	[93]
Myrcia splendens (Sw.) DC.	L	HEth	MIC = 250 µg/mL	*Escherichia coli*	Broth Microdilution Assay	*E. coli* (ATCC 11775)	[93]
Psidium cattleyanum Sabine	L	PL	MIC = 1.56-3.13 mg/mL	*Staphylococcus aureus, Listeria monocytogenes, Pseudomonas aeruginosa* and *Enterococcus faecalis*	Agar Diffusion Assay	*S. aureus* (ATCC 25923), *L. monocytogenes Scott A, P. aeruginosa* (ATCC 9027) and *E. faecalis* FAIR (E77)	[94]
Psidium guineense Sw.	L	EO	MIC = 126.4 and 231.9 µg/mL	*Mycobacterium tuberculosis*	REMA method	*M. tuberculosis virulent type (H37Rv)*	[95]
Psidium myrsinites DC.	L	C	MIC = 62.5 and 125 µg/mL	*Staphylococcus epidermidis and Staphylococcus aureus*	Broth Microdilution Assay	*S. epidermidis* (ATCC 12228) and *S. aureus* (ATCC 25923)	[96]
Campomanesia adamantium (Cambess.) O.Berg	F and L	EA and Eth	MIC = 62.5 µg/mL	*Mycobacterium tuberculosis, Staphylococcus aureus, Staphylococcus epidermidis* and *Enterobacter cloacae*	Microplate Alamar Blue and Broth Microdilution Assay	*M. tuberculosis H37Rv* (ATCC 27294), *S. aureus* (ATCC 25923), *S. epidermidis* (ATCC 12229) *and E. cloacae*	[97,98]
Campomanesia sessiliflora (O.Berg) Mattos	L	EO	MIC = 31.25 µg/mL	*multi−resistant Staphylococcus sp. 841*	Broth Microdilution Assay	multi−resistant *Staphylococcus sp. 841*	[70]
**Phytolaccaceae**							
Gallesia integrifolia (Spreng.) Harms	B	HEth	MIC = 25-400 µg/mL and 10-250 mg/kg p.o	*Shigella flexneri, Streptococcus pyogenes, Escherichia coli, Salmonella typhimurium, Klebsiella pneumoniae, Pseudomonas aeruginosa* and *Staphylococcus aureus*	Broth Microdilution Assay	*S. flexneri, S. pyogenes, E. coli, S. typhimurium, K. pneumonia, P.aeruginosa and S. aureus strains, S. aureus* strain inoculated in female albino Wistar rats	[99]

**Primulaceae**							
Myrsine guianensis (Aubl.) Kuntze	L	EO	MIC = 31.25 μg/mL	*multi−resistant Staphylococcus sp. 841*	Broth Microdilution Assay	multi−resistant *Staphylococcus sp. 841*	[70]
**Proteaceae**							
Roupala montana var. brasiliensis (Klotzsch) K.S.Edwards	SB and L	Eth and W	MIC = 5 and 125 μg/mL	*Staphylococcus aureus* and *Escherichia coli*	Broth Microdilution and Agar Diffusion Assay	*S. aureus* (ATCC 25923) and *E. coli*	[79,82]
**Sapindaceae**							
Serjania marginata Casar.	L	HA	MIC= 75-250 μg/mL	*Helicobacter pylori*	Broth Microdilution Assay	*H. pylori* (ATCC 43504)	[100]
**Siparunaceae**							
Siparuna guianensis Aubl.	L	EO	MIC = 50-500 μg/mL	*Streptococcus mutans, Streptococcus mitis, Streptococcus sobrinus, Streptococcus salivarius, Mycobacterium tuberculosis, Mycobacterium kansasii,* and *Mycobacterium avium*	Broth Microdilution Assay	*S. mutans* (ATCC 25175); *S. mitis* (ATCC 49456); *S. salivarius* (ATCC 25975) and *S. sobrinus* (ATCC 33478); *M. tuberculosis H37Rv* (ATCC 27294) and *M. kansasii* (ATCC 12478) and *M. avium* (ATCC 25291)	[101]
**Vochysiaceae**							
Qualea parviflora Mart.	B	M	MIC = 75 μg/mL	*Helicobacter pylori*	Broth Microdilution Assay	*H. pylori* (ATCC 43504)	[102]

Part of plant[1] (AP= aerial parts; F= Fruits; FL= Flowers; L= Leaves; S= Stem; Stem Bark= SB; Bark= B; Whole plant= WP; Pulp= P; Xylopodium= X).
Type of extract[2] (Acetogenin-rich fraction= AC; Aqueous= Aq; Dichloromethane= DM; Ethanolic= Eth; Ethyl-acetate= EA; Hexane= HE; Essential oils= EO; Fixed oil= FO; Crude= C; Methanol= M; Hydroalcoholic= HA; Stalk's inner bark= SIB; Hydroethanolic= HEth; Brazilian cachaça was used as the extractor liquid = BC; Dry extract = D; Water extracts = W; Powder extract = Pw; Pressurized liquid extraction with water= PL).

Table S5 Antifungal activity from different species of Cerrado. Information about part from plant and extract preparation, dose tested, model and method analyzed.

Family/Species	Part[1]	Extract[2]	Dose/Concentration	Activity against	Technique	Model	Reference
**Anacardiaceae**							
Myracrodruon urundeuva M. Allemão	IB	Aq	MIC = 31.25 μg/mL	*Candida albicans*	Broth Microdilution Assay	*C. albicans* isolated from the oral mucosa of individuals with HIV/AIDS	[103]
**Annonaceae**							

Cardiopetalum calophyllum Schltdl.	L	EO	300 µl/mL	*Sclerotinia sclerotiorum*	Disk Diffusion Test	*S. sclerotiorum*	[62]
**Arecaceae**							
**Asteraceae**							
Mikania glomerata Spreng	L	EO	MIC = 0.25 mg/mL	*Candida albicans*	Minimal Inhibitory Concentration (MIC) Test	*C. albican*s CBMAI 0475 (ATCC 10231)	[104]
**Bignoniaceae**							
Pyrostegia venusta (Ker Gawl.) Miers	FL	n-BF	MIC= 0.7-6.0 µg/mL	*Candida albicans* and *clinical*, *Candida krusei ,Candida tropicalis, Candida parapsilosis* and *Candida guilhermondii*	Broth Microdilution Assay	*C. albicans* (ATCC 10231), C. *albicans* (USP 1), *C. albicans* (USP-1565), *C. albicans* (OF M3-20), *C. albicans* (OF M7-19), *C. krusei* (ATCC 6258), *C. krusei* (USP-2223), *C. tropicalis* (USP-B3), *C. tropicalis* (USP-1658), *C.parapsilosis* (USP-1933), and *C. guilhermondii* (USP)	[105]
**Bixaceae**							
Cochlospermum regium (Mart. ex Schrank) Pilg.	R and L	EA and Eth	MIC= 31.25-250 µg/mL and 0.5 mg/mL	*Candida albicans, Candida krusei, Candida glabrata, Candida tropicalis* and *Candida*	Broth Microdilution Assay	*C. albicans* (10231 250), *C. krusei* (34135), *C. glabrata* (13813), *C. tropicalis* (ATCC 750) and *C. tropicalis* clinical isolated yeasts	[71,106]
**Calophyllaceae**							
Calophyllum brasiliense Cambess.	RW	DM	MIC= 1.95 and 1.95 µg/mL	*Cryptococcus gattii, Candida albicans* and *Candida krusei*	Broth Microdilution Assay	*C. gattii* (LMGO 01), *C. albicans* (ATCC 10231) and *C. krusei* (LMGO 174)	[107]
**Combretaceae**							
Terminalia fagifolia Mart.	SB	Aq	MIC = 0.4-25 µg/mL	*Candida albicans, Candida tropicalis,*	Broth Microdilution Assay	*C. albicans* (SC 5314/ATCC MYA-	[108]

				Candida parapsilosis and Candida albicans		2876), *C. tropicalis* (ATCC 750), *C. parapsilosis* (ATCC 22901) and *C. albicans* (ATCC 96901 fluconazole-resistant strain)	
**Connaraceae**							
Connarus suberosus Planch.	RB	Eth	MIC= 15.62-250 µg/mL	*Candida albicans, Candida parapsilosis* and *Candida glabrata*	Broth Microdilution Assay	*C. albicans* (ATCC 10231), *C. parapsilosis* (ATCC 22019) and *C. glabrata* (LMGO 44)	[109]
**Dilleniaceae**							
Curatella americana L.	SB and B	C, BC	MIC= 7.8-125 µg/mL	*Candida albicans, Candida tropicalis, Candida parapsilosis, Candida albicans*	Broth Microdilution Assay	*C. albicans* (LC352), *C. tropicalis* (LC299), *C. parapsilosis* (LC144), *C. parapsilosis, C. tropicalis* (four strains that were resistant to fluconazole, *C. albican*s 32res, 32Bres, 48res, and 103res), (*C. albicans* 48sen), *C. albicans* (ATCC 10231) and *C. parapsilosis* (ATCC 22019)	[75,110]
**Erythroxylaceae**							
Erythroxylum suberosum A.St.-Hil.	SB	EA	MIC= 62.5-500 µg/mL	*Candida krusei* and *Cryptococcus neoformans*	Broth Microdilution Assay and Disk Diffusion Test	*C. krusei* (ATCC 6258) and *C. neoformans* (ATCC 32045)	[79]
**Fabaceae**							
Tachigali aurea Tul.	RW and B	EA and BC	MIC= 0.12-125 µg/mL	*Candida albicans, Candida parapsilosis, Candida glabrata, Trichophyton*	Broth Microdilution Assay	*C. albicans* (ATCC 10231), *C. parapsilosis*(ATCC 22019), *C. glabrata* (LMGO 44), *T.*	[75,111]

				mentagrophytes and *Trichophyton rubrum*		*mentagrophytes* (LMGO 09) and *T. rubrum* (LMGO 06)	
Hymenaea martiana Hayne	T and B	C	MIC= 4-64 µg/mL	*Cryptococcus neoformans, Cryptococcus gattii, Trichopyton rubrum, Trichopyton mentagrophytes* and *Microsporum canis*	Broth Microdilution Assay	*C. neoformans, C. gattii T. rubrum, T. mentagrophytes* and *M. canis*	[112]
Plathymenia reticulata Benth.	B	BC	MIC= 62.5 µg/mL	*Candida albicans* and *Candida parapsilosis*	Broth Microdilution Assay	*C. albicans* (ATCC 10231) and *C. parapsilosis* (ATCC 22019)	[75]
Vatairea macrocarpa (Benth.) Ducke	RB	EA	MIC= 0.98- 125 µg/mL	*Candida albicans, Candida parapsilosis, Candida, glabrata, Trichophyton mentagrophytes* and *Trichophyton rubrum*	Broth Microdilution Assay	*C. albicans* (ATCC 10231), *C. parapsilosis* (ATCC 22019), *C. glabrata* (LMGO 44), *T. mentagrophytes* (LMGO 09) and *T. rubrum* (LMGO 06)	[111]
Copaifera langsdorffii Desf.	S	OR	MIC= 62.5 µg/mL	*Paracoccidioides lutzii, Paracoccidioides brasiliensis, Paracoccidioides americana* and *Paracoccidioides restrepiensis*	Broth Microdilution Assay	*P. lutzii* (Pb01), *P. brasiliensis* (Pb18), *P. americana* (Pb03), and *P. restrepiensis* (EPM83)	[113]
Inga laurina (Sw.) Willd.	L	Eth	MIC= 11.7-93.8 µg/mL	*Candida albicans, Candida glabrata* and *Candida tropicalis*	Broth Microdilution Assay	*C. albicans* (ATCC 28366), *C. glabrata* (ATCC 15126) and *C. tropicalis* (ATCC 13803)	[114]
Stryphnodendron adstringens (Mart.) Coville	L	D	MIC= 12.5 µg/mL	*Candida albicans, Candida tropicalis* and *Candida parapsilosis*	Broth Microdilution Assay	*C. albicans* (ATCC 18804), *C. tropicalis* (ATCC 13803) and *C. parapsilosis* (ATCC 22009)	[83]

**Lamiaceae**							
Hyptis crenata Pohl ex Benth.	WP	Eth	MIC= 125 µg/mL	*Candida krusei* and *Cryptococcus neoformans*	Broth Microdilution Assay	*C. krusei* (ATCC 6258) and *C. neoformans* (ATCC 32045)	[79]
**Lythraceae**							
Lafoensia pacari A.St.-Hil.	SB	EA and Eth	MIC= 100-1000 µg/mL	*Candida krusei,Candida parapsilosis, Saccharomyces cerevisiae and Cryptococcus neoformans*	Broth Microdilution Assay	*C. krusei* (ATCC 6258), *C. parapsilosis* (ATCC 22019), *S. cerevisiae* (ATCC 9763) and *C. neoformans* (ATCC 32264)	[115]
**Malpighiaceae**							
Banisteriopsis argyrophylla (A.Juss.) B.Gates	L	Eth	MIC =31.25-93.75 µg/mL	*Candida albicans, Candida tropicalis* and *Candida glabrata*	Broth Microdilution Assay	*C. albicans* (ATCC 28366), *C. tropicalis* (ATCC 13803) and *C. glabrata* (ATCC 15126)	[116]
Banisteriopsis laevifolia (A.Juss.) B.Gates	L	Eth	MIC= 31 and 63 µg/mL	*Candida albicans, Candida tropicalis and Candida glabrata*	Broth Microdilution Assay	*C. albicans* (ATCC28366), *C. tropicalis* (ATCC13803) and *C. glabrata* (ATCC15126)	[117]
**Moraceae**							
Brosimum gaudichaudii Trécul	R	DM	1 mg	*Cladosporium sphaerospermum*	Bioautographic Assay	Spore suspension of the fungus *C. sphaerospermum*	[82]
**Myrtaceae**							
Campomanesia adamantium (Cambess.) O.Berg	L	Eth	MIC = 7.81 and 31.25 µg/mL	*Candida krusei, Candida tropicalis, Cryptococcus neoformans var. neoformans* and *Cryptococcus neoformans var. gatti*	Broth Microdilution Assay	*C. krusei* (ATCC 34135), *C. tropicalis* (ATCC 28707), *C. neoformans* var. *neoformans* (L2) and *C.*	[97]

						neoformans var. *gatti* (L1)	
Eugenia dysenterica (Mart.) DC.	L	Aq and EO	MIC= 125-500 µg/disc and 15.6-250 µg/mL	*Candida guilliermondii* , *Candida tropicalis* , *Candida parapsilosis* , *Candida famata, Candida krusei, Cryptococcus neoformans var.*		*C. guilliermondii* (ATCC 6260), *C. tropicalis* (ATCC 28707), *C.parapsilosis* (ATCC 22019), *C. famata* (ATCC 62894),*C. krusei* (ATCC 34135), *C. neoformans* var. *gattii* and *C. neoformans* var.	[118,119]
Eugenia involucrata DC.	L	HE	MIC= 31.2-62.5 µg/mL	*Cryptococcus sp. D, Cryptococcus gatti* and *Cryptococcus neoformans*	Broth Microdilution Assay	*Cryptococcus* sp. D, *C.gatti* (L48), and *C.neoformans* (L3)	[120]
Myrcia linearifolia Cambess	R	EA	MIC= 31.15-125 µg/mL	*Candida albicans, Candida parapsilosis* and *Candida glabrata*	Broth Microdilution Assay	*C. albicans* (ATCC 10231), *C. parapsilosis* (ATCC 22019) and *C. glabra*ta (LMGO 44),	[121]
Psidium brownianum Mart. ex DC.	L	Aq	MIC = 2.05-8.192 µg/mL µg/mL	*Candida albicans* and *Candida tropicalis*	Broth Microdilution Assay	clinical isolates of *C. albicans* and *C. tropicalis* namely (CA INCQS 40006, CA LM 77, CT INCQS 40042 and CT LM 23)	[122]
**Nyctaginaceae**							
Neea theifera Oerst.	SW	HE	MIC = 0.122-62.5 µg/mL	*Candida albicans, Candida parapsilosis* and *Candida glabrata*	Broth Microdilution Assay	*C. albicans* (ATCC 10231), *C. parapsilosis* (ATCC 22019) and *C. glabra*ta (LMGO 44)	[121]
**Proteaceae**							
Roupala montana var. brasiliensis (Klotzsch) K.S.Edwards	SB	Eth	MIC =15.6-500 µg/mL	*Candida albicans, Candida glabrata, Candida krusei, Candida parapsilosis, Candida*	Broth Microdilution Assay	*C. albicans* (ATCC 90028), *C. glabrata* (ATCC 9030), *C. krusei* (ATCC 6258), *C.*	[79]

				tropicalis and *Cryptococcus neoformans*		*parapsilosis* (ATCC 22019), *C. tropicalis* (ATCC 760) and *C. neoformans* (ATCC 32045)	
**Rutaceae**							
Spiranthera odoratissima A.St.-Hil.	L	EA	MIC= 31.25 µg/mL	*Cryptococcus gattii*	Broth Microdilution Assay	*C.gattii* (LMGO 01)	[107]
**Sapindaceae**							
Matayba guianensis Aubl.	RB	Eth	MIC= 0.97-31.25 µg/mL	*Candida albicans, Candida parapsilosis, Trichophyton mentagrophytes* and *Trichophyton rubrum*	Broth Microdilution Assay	*C. albicans* (ATCC 10231), *C. parapsilosis* (ATCC 22019), *T. mentagrophytes* (LMGO 09) and *T. rubrum* (LMGO 06)	[123]
**Sapotaceae**							
Pouteria ramiflora (Mart.) Radlk.	L	Aq	MIC = 500 µg/disc	*Candida tropicalis*	Disk Diffusion Test	*C. tropicalis* (ATCC 28707)	[118]

Part of plant[1] (FL= Flowers, L= Leaves; Stem Bark= SB; Whole plant= WP; Inner bark = IB; Stem= S; Stem wood = SW; Root Wood= RW; RB=root bark; R= Root).
Type of extract[2] (Aqueous= Aq; Dichloromethane= DM; Ethanolic= Eth; Ethyl-acetate= EA; Essential oils= EO; Brazilian cachaça was used as the extractor liquid = BC; Dry extract = D; Inner bark = IB; Resin oil = OR; Dry extract = D; n-butanol= n-BF; Hexane= HE).

Table S6 Antiviral activity from different species of Cerrado. Information about part from plant and extract preparation, dose tested, model and method analyzed.

Family/Species	Part[1]	Extract[2]	Dose/Concentration	Activity against	Technique	Model	Reference
**Anacardiaceae**							
Myracrodruon urundeuva M. Allemão	L	Eth	50 and 500 µg/mL	Simian rotavirus SA11	Rotavirus cytopathic effect inhibition and confirmation by RT-PCR	MA-104 cells	[124]
**Annonaceae**							
Anaxagorea dolichocarpa Sprague & Sandwith	S	Eth	EC50 = 90.5 µg/mL	EMCV	Cytopathic effect inhibition	Vero cells	[125]
Annona dolabripetala Raddi	L	Eth	EC50 = 13.3 µg/mL	VACV	Cytopathic effect inhibition	Vero cells	[125]
**Apocynaceae**							
Aspidosperma tomentosum Mart. & Zucc.	L	DM	IC50 = 45.86 µg/mL	Avian metapneumovirus	Cytopathic effect inhibition	Chicken embryo related cell	[126]
Hancornia speciosa Gomes	S	Eth	> EC50 > 81.9 µg/mL	HSV-1, VACV, and EMCV	Cytopathic effect inhibition	Vero cells	[125]
Himatanthus phagedaenicus (Mart.) Woodson	S	Eth	EC50= 48.2 µg mL	HSV-1	Cytopathic effect inhibition	Vero cells	[125]
**Bignoniaceae**							
Amphilophium elongatum (Vahl) L.G.Lohmann	L or S	Eth	EC50 > 24.3 µg/ mL	VACV	Cytopathic effect inhibition	Vero cells	[127]
Cuspidaria sceptrum (Cham.) L.G.Lohmann	S	Eth	EC50= 40.6 µg/ mL	VACV	Cytopathic effect inhibition	Vero cells	[128]
Fridericia formosa (Bureau) L.G.Lohmann	L or S or F	Eth	EC50 > 148.5 µg/mL	HSV-1	Cytopathic effect inhibition	Vero cells	[128]
Fridericia samydoides (Cham.) L.G.Lohmann	L or S	Eth	EC50 > 377.2 µg/mL	HSV-1, VACV, and EMCV	Cytopathic effect inhibition	Vero cells	[127]
Zeyheria tuberculosa (Vell.) Bureau ex Verl.	L	Eth	EC50 > 81.8 µg/mL	HSV-1 and VACV	Cytopathic effect inhibition	Vero cells	[128]
Anemopaegma setilobum A.H.Gentry	S	Eth	EC50 = 95.2 µg/mL	EMCV	Cytopathic effect inhibition	Vero cells	[128]
Callichlamys latifolia (Rich.) K.Schum.	L or S	Eth	EC50 > 312.3 µg/mL	HSV-1, VACV, and EMCV	Cytopathic effect inhibition	Vero cells	[127]

Fridericia chica (Bonpl.) L.G.Lohmann	L	M	IC50 = 412.7 µg/mL	Avian metapneumovirus	Cytopathic effect inhibition	chicken embryo related cell	[126]
Fridericia craterophora (DC.) L.G.Lohmann	L	Eth	EC50 = 68.2 µg/mL	EMCV	Cytopathic effect inhibition	Vero cells	[128]
**Dilleniaceae**							
Curatella americana L.	B	BC	EC50 = 49 µg/mL	Poliovirus	Cytopathic effect inhibition	Vero cells	[75]
**Ericaceae**							
Gaylussacia brasiliensis (Spreng.) Meisn. var. brasiliensis	L	DM	IC50 = 22.33 µg/mL	Avian metapneumovirus	Cytopathic effect inhibition	Chicken embryo related cell	[126]
**Fabaceae**							
Hymenaea courbaril L.	L	Eth	500 µg/mL	Simian rotavirus SA11	Rotavirus cytopathic effect inhibition and confirmation by RT-PCR	MA-104 cells	[129]
Bauhinia holophylla (Bong.) Steud.	L	HEth	IC50= 3.2 µg/mL	DENV-2	Cytopathic effect inhibition	Vero cells	[130]
**Lamiaceae**							
Hyptis radicans (Pohl) Harley & J.F.B.Pastore	AP	Eth, HE, and HM	MIC50= 159 and 180 µg/mL	HIV-1	Reverse transcriptase (RT) inhibitory assay	*in vitro*	[84]
**Malpighiaceae**							
Byrsonima verbascifolia (L.) DC.	L	Eth	500 µg/mL	Simian rotavirus SA11	Rotavirus cytopathic effect inhibition and confirmation by RT-PCR	MA-104 cells	[129]
**Myristicaceae**							
Virola sebifera Aubl.	L	DM	IC50 = 202 µg/mL	Avian metapneumovirus	Cytopathic effect inhibition	Chicken embryo related cell	[126]
**Myrtaceae**							
Eugenia dysenterica (Mart.) DC.	L	Eth	500 µg/mL	Simian rotavirus SA11	Rotavirus cytopathic effect inhibition and confirmation by RT-PCR	MA-104 cells	[129]

**Ochnaceae**							
Ouratea spectabilis (Mart.) Engl.	L	Eth	EC50 > 11.4 µg/mL	HSV-1 and VACV	Cytopathic effect inhibition	Vero cells	[125]
Ouratea castaneifolia (DC.) Engl.	L	Eth	EC50 > 465.7 µg/mL	HSV-1, VACV, and EMCV	Cytopathic effect inhibition	Vero cells	[125]
Ouratea semiserrata (Mart. & Nees) Engl.	L or S	Eth	EC50 > 254.4 µg/mL	HSV-1, VACV, and EMCV	Cytopathic effect inhibition	Vero cells	[125]
**Polygonaceae**							
Polygonum spectabile Mart.	AP	Eth	EC50 > 30.5 µg/mL	HSV-VACV	Cytopathic effect inhibition	Vero cells	[125]
**Vitaceae**							
Cissus erosa Rich.	S	Eth	EC50 = 27.9 µg/mL	VACV	Cytopathic effect inhibition	Vero cells	[125]

Part of plant[1] (L= Leaves; S= Stem; F= Fruits; Bark= B; AP= aerial parts).
Type of extract[2] (Dichloromethane= DM; Ethanolic= Eth; Ethyl-acetate= EA; Hexane= HE; Methanol= M; Ethanolic= Eth; Hydromethanolic=HM).

Table S7 Antiparasitic activity from different species of Cerrado. Information about part from plant and extract preparation, dose tested, model and method analyzed.

Family/Species	Part[1]	Extract[2]	Dose /Concentration	Activity against	Technique	Experimental model	Reference
**Annonaceae**							
Annona coriacea Mart.	L	BC	IC50 = 175 μg/mL	*Leishmania amazonensis*	N/S	Promastigotes	[75]
Annona crassiflora Mart.	RB	Eth	IC50= 3.7 and 5.9 μg/mL	*Leishmania donovani* and *Trypanosoma cruzi*	MTT assay and Growth inhibition	Promastigotes	[131]
Duguetia furfuracea (A.St.-Hil.) Saff.	RB	HE	IC50 = 6.6 μg/mL	*Trypanosoma cruzi*	Growth inhibition	Amastigotes	[131]
Xylopia aromatica (Lam.) Mart.	RW	HE	IC50 = 4.7 μg/mL	*Plasmodium falciparum*	Tritiated hypoxanthine incorporation assay	Chloroquine-resistant strain	[132]
Xylopia emarginata Mart.	RB	HE	IC50 = 4.9 μg/mL	*Plasmodium falciparum*	Tritiated hypoxanthine incorporation assay	Chloroquine-resistant strain	[132]
**Apocynaceae**							
Aspidosperma tomentosum Mart. & Zucc.	S	Eth	IC50 = 9.70 and 15.88 μg/mL	*Leishmania infantum*	Resazurin and infection of macrophage cultures method	Promastigotes and amastigotes	[133]
Himatanthus obovatus (Müll. Arg.) Woodson	B	Eth	IC50 = 7.5 μg/mL	*Leishmania donovani*	MTT assay	Promastigotes	
**Aspidosperma**							
Aspidosperma macrocarpon Mart. & Zucc.	RB	Eth	IC50 = 4.9 μg/mL	*Plasmodium falciparum*	Tritiated hypoxanthine incorporation assay	Chloroquine-resistant strain	[132]
**Asteraceae**							
Cyrtocymura scorpioides (Lam.) H.Rob.	AP	EA	IC50 = 16.0 μg/mL	*Leishmania amazonensis*	Infection of macrophage cultures	Amastigotes	[134]
Eremanthus incanus (Less.) Less.	AP	EA	IC50 = 6.0 μg/mL	*Leishmania amazonensis*	Infection of macrophage cultures	Promastigotes	[134]
Pseudogynoxys cabrerae H.Rob. & Cuatrec.	AP	EA	IC50 = 5.0 μg/mL	*Leishmania amazonensis*	Infection of macrophage cultures	Tmastigotes	[134]
**Bignoniaceae**							
Jacaranda caroba (Vell.) DC.	L	Eth	IC50 = 13.22 μg/mL	*Leishmania amazonensis*	MTT assay	promastigotes	[135]
Jacaranda cuspidifolia Mart.	L	Eth	IC50 = 10.96 μg/mL	*Leishmania amazonensis*	MTT assay	Promastigotes	[135]
**Caryocaraceae**							

Caryocar coriaceum Wittm.	F or L	Eth and EA	IC50 < 38 µg/mL	*Leishmania amazonensis*	Antipromastigote assay	Promastigotes	[136,137]
**Celastraceae**							
**Connaraceae**							
Connarus suberosus Planch.	RB or RW	HE	IC50 = 1.8 27.57 µg/mL	*Leishmania amazonensis, Trypanosoma brucei gambiense* and *Plasmodium falciparum*	MTT assay	Promastigotes	[135,138]
**Ebenaceae**							
Diospyros hispida A.DC.	R	EA	IC50 = 1 and 18.9 µg/mL	*Leishmania chagasi* and *Plasmodium falciparum*	MTT and Tritiated hypoxanthine incorporation assay	Promastigotes and chloroquine-resistant strain	[107]
Diospyros lasiocalyx (Mart.) B.Walln.	L	Eth	IC50 = 55.48 and 80.63 µg/mL	*Leishmania infantum*	Resazurin method and Infection of macrophage cultures	Promastigotes and amastigotes	[133]
**Euphorbiaceae**							
Croton urucurana Baill.	SW	HE	IC50 = 3.5 µg/mL	*Plasmodium falciparum*	Tritiated hypoxanthine incorporation assay	Chloroquine-resistant strain	[107]
**Fabaceae**							
Dipteryx alata Vogel	L	HE	IC50 = 0.08 µg/mL	*Leishmania amazonensis*	MTT assay	Promastigotes	[135]
Enterolobium gummiferum (Mart.) J.F.Macbr.	SB	EA	IC50 = 9.23 µg/mL	*Leishmania amazonensis*	MTT assay	Promastigotes	[139]
Hymenaea courbaril L.	L	Eth and HE	IC50 < 44.10 µg/mL	*Leishmania amazonensis*	MTT assay	Promastigotes	[135]
Hymenaea stigonocarpa Mart. ex Hayne	L	Eth	IC50 = 4.69 µg/mL	*Leishmania amazonensis*	MTT assay	Promastigotes	[135]
Lachesiodendron viridiflorum (Kunth) P.G. Ribeiro, L.P. Queiroz & Luckow	L	Aq and Eth	IC90 = 2.1 and 2.4 µg/mL and 283 mg/kg bw	*Haemonchus contortus*	Egg hatching and fecal egg count inhibition test	Fresh nematode eggs and lambs infected with H. contortus L3	[140]
Stryphnodendron rotundifolium Mart.	SB	Or	IC50 = 1.7- 24.5 µg/mL	*Leishmania amazonensis*	MTT assay	Promastigotes	[141]
Vatairea macrocarpa (Benth.) Ducke	RB	EA	IC50 = 71.47 µg/mL	*Leishmania amazonensis*	MTT assay	Promastigotes	[139]
**Lauraceae**							

Nectandra megapotamica (Spreng.) Mez	L	EO	IC50 = 98.7 µg/mL	*Trichomonas vaginalis*	Trypan blue assay	Trophozoites	[142]
**Malvaceae**							
Guazuma ulmifolia Lam	L	Eth	EC50 = 282.63-454.68 µg/mL	*Leishmania brasiliensis, Leishmania infantum* and *Trypanosoma cruzi*	Chlorophenol red-b-Dgalactopyranoside assay	Promastigotes and epimastigotes	[143]
**Melastomataceae**							
Pleroma stenocarpum (Schrank et Mart. ex DC.) Triana	AP	HE	IC50 = 23.6 µg/mL	*Trypanosoma cruzi*	N/S	Trypomastigotes	[144]
**Meliaceae**							
Guarea kunthiana A.Juss.	R	HE	IC50 = 7.9 µg/mL	*Leishmania donovani*	MTT assay	Promastigotes	[131]
**Myrtaceae**							
Blepharocalyx salicifolius (Kunth) O.Berg	L or SB	He, Eth and EA	IC50 < 7 9.3 µg/mL	*Trypanosoma brucei gambiense* and *Plasmodium falciparum*	N/S	Bloodstream forms and chloroquine-resistant strain	[145]
Eugenia uniflora L.	L	EO	IC50 = 0.99 µg/mL	*Leishmania amazonensis*	flagellar motility avaliation	Promastigotes	[39]
Myrcia variabilis DC.	AP	Eth	IC50 = 30.5 µg/mL	*Trypanosoma cruzi*	N/S	Trypomastigotes	[144]
Psidium brownianum Mart. ex DC	L	HEth or Aq	1000 µg/mL	*Trypanosoma cruzi*	Chlorophenol red-b-Dgalactopyranoside assay	Tpimastigotes	[41]
Psidium laruotteanum Cambess.	L	HE or EA	IC50 < 6.8 µg/mL	*Trypanosoma brucei gambiense*	N/S	Bloodstream forms	[145]
**Primulaceae**							
Myrsine guianensis (Aubl.) Kuntze	SW	HE	IC50 = 5.0 µg/mL	*Plasmodium falciparum*	N/S	Chloroquine-resistant strain	[138]
**Rubiaceae**							
Genipa americana L.	L	M	IC50 = 470 and 710 µg/mL	*Trypanosoma cruzi*	Cell infection and mobility avaliation	Amastigotes, epimastigotes and trypomastigotes	[92]
Spiranthera odoratissima A.St.-Hil.	R and L	HE and EA	IC50 = 22.3 and 56.3 µg/mL	*Leishmania chagasi* and *Trypanosoma cruzi*	MTT assay	Epimastigotes and promastigotes	[107]

**Salicaceae**							
Casearia sylvestris Sw. var. sylvestris	L, RB, SW, SB or RW	Eth or HE	IC50 < 9.48 μg/mL	*Leishmania donovani*, *Plasmodium falciparum*, *Leishmania infantum* and *Trypanosoma cruzi*	Growth inhibition, tritiated hypoxanthine incorporation and MTT assay	Amastigotes, chloroquine-resistant strain promastigotes andtrypomastigotes	[131,132,146]
**Sapindaceae**							
Serjania lethalis A.St.-Hil.	RB, L and S	HE and DM	IC50 < 28.33 μg/mL	*Leishmania donovani* and *Leishmania amazonensis*	MTT, XTT assay and infection of macrophage cultures	Promastigotes and amastigotes	[131,147]
Cupania vernalis Cambess.	L	HE	IC50 = 0.9 and 7.1 μg/mL	*Leishmania donovani* and *Plasmodium falciparum*	MTT and tritiated hypoxanthine incorporation assay	Promastigotes and chloroquine-resistant strain	[131,132]
Simarouba versicolor A.St.-Hil.	L	DM	IC50 = 3.1 μg/mL	*Plasmodium falciparum*	Tritiated hypoxanthine incorporation assay	chloroquine-resistant strain	[107]
**Solanaceae**							
Solanum palinacanthum Dunal	F	Eth	IC50 = 15.3 and 175.9 μg/mL	*Trypanosoma cruzi*	MTT assay	Epimastigotes	[148]
Solanum lycocarpum A.St.-Hil.	F	Eth or HA	IC50 = 3.0-194.7 μg/mL	*Leishmania infantum* and *Trypanosoma cruzi*	flagellar motility avaliation, infection of macrophage cultures and MTT assay	Promastigotes, amastigotes, epimastigotes and trypomastigotes	[144,148,149]
**Vochysiaceae**							
Qualea grandiflora Mart.	L and F	Eth and EA	IC50 < 15 μg/mL	*Trypanosoma brucei gambiense* and *Plasmodium falciparum*	Resazurin method and SYBR Green I method	Bloodstream forms and sensitive and resistant strains	[150,151]

Part of plant[1] (AP= aerial parts; F= Fruits; L= Leaves; RB=root bark; R= Root; Root Wood= RW; S= Stem; Stem Bark= SB; Bark= B).
Type of extract[2] (Aqueous= Aq; Dichloromethane= DM; Ethanolic= Eth; Ethyl-acetate= EA; Hexane= HE; Essential oils= EO; Hydroalcoholic= HÁ; Hydroethanolic= HEth; Organic fraction=Or; Methanol= M).
NOT SPECIFIED=NS

Table S8 Insecticidal activity from different species of Cerrado. Information about part from plant and extract preparation, dose tested, model and method analyzed.

Family/Species	Part[1]	Extract[2]	Dose /Concentration	Activity against	Technique	Model	Reference
**Anacardiaceae**							
Anacardium occidentale L.	N	EO	LC50 = 14.5 µg/L	*Aedes aegypti*	Mortality determination	4th-stage larvae	[152]
**Annonaceae**							
Annona crassiflora Mart.	R and S	Eth and Chl	LC50 = 23.06 µg/mL	*Aedes aegypti* and *Chrysodeixis includens*	Mortality determination and topical application	3rd-stage larvae and larvae	[28, 229]
Annona mucosa Jacq.	S	Eth	411.55 mg/kg	*Helicoverpa armigera*	Greenhouse trial	neonate larvae	[155]
Duguetia furfuracea (A. St. Hil.) Benth & Hook	R	HE	LC50 = 56.6 µg/mL	*Aedes aegypti*	Mortality determination	3rd-stage larvae	[153]
Duguetia lanceolata A.St.-Hil.	L	EO	LC50 = 442 and 457 µg/mL	*Zabrotes subfasciatus* and *Sitophilus zeamais*	Residual contact	0 to 24 h aged insects and nonsexed 10 to 20-day-old adults	[156]
Xylopia aromatica (Lam.) Mart.	L or F	Eth and EO	LC50 = 384.37 µg/mL	*Aedes aegypti* and *Bemisia tabaci*	Mortality determination and no-choice and free-choice bioassay	3rd-stage larvae and adults whiteflies	[153,157]
**Apocynaceae**							
Aspidosperma macrocarpon Mart. & Zucc.	RW	HE	IC50 > 1000 µg/mL	*Zabrotes subfasciatus*	*In vitro* inhibition assay	Larvae digestive enzymes	[158]
**Asteraceae**							
Ageratum conyzoides L.	L	EO	LC50 = 148 µg/L	*Aedes aegypti*	Mortality determination	4th-stage larvae	[152]
Ageratum fastigiatum (Gardner) R.M.King & H.Rob.	L and FL	Eth	Mortality = 6.67%	Coccinellidae predators	Residual contact	4-day-old adult	[159]
Piptocarpha rotundifolia (Less.) Baker	R	HE	LC50 = 162.31 µg/mL	*Aedes aegypti*	Mortality determination	3rd-stage larvae	[153]
**Bignoniaceae**							

Adenocalymma nodosum (Silva Manso) L.G.Lohmann	L	MA	80% mortality at 0.01%	Tenebrio molitor	Topical application	2-day-old pupae	[160]
Cybistax antisyphilitica (Mart.) Mart.	S	HE	LC50 = 27.61 µg/mL	*Aedes aegypti*	Mortality determination	3rd-stage larvae	[153]
**Calophyllaceae**							
Kielmeyera coriacea Mart. & Zucc.	SB	Heth	IC50 = 110 and 272.12 µg/mL	*Zabrotes subfasciatus* and *Acanthoscelides obtectus*	*In vitro* inhibition assay	Larvae digestive enzymes	[158]
**Combretaceae**							
Terminalia fagifolia Mart.	S	Eth	LC50 = 373.14 µg/mL	*Aedes aegypti*	Mortality determination	Larvae	[161]
**Fabaceae**							
Anadenanthera colubrina (Vell.) Brenan	W	CHE	LC50 = 11.9 µg/mL	*Nasutitermes corniger*	No-choice feeding termiticidal assay	active termites	[162]
Bowdichia virgilioides Kunt	HW	CHE	LC50 = 7.2 or 21.95 µg/mL	*Nasutitermes corniger* and *Aedes aegypti*	Mortality determination and No-choice feeding termiticidal assay	4th-stage larvae and active termites	[162,163]
Copaifera multijuga Hayne	OR and B	EO, Eth	LC50 = 0.7, 0.9, 3 and 18 ppm	*Aedes aegypti* and *Anopheles darlingi*	Mortality determination	larvae	[164]
Hymenaea stigonocarpa Mart. ex Hayne	HW	EA	LC50 = 17.3 µg/mL	*Nasutitermes corniger*	No-choice feeding termiticidal assay	Active termites	[162]
Copaifera langsdorffii Desf.	S, L and F	EO and M	LC50 = 13.7-41 µg/mL	*Aedes aegypti* and *Spodoptera frugiperda*	Mortality determination and artificial diet	4th-stage larvae and 1st and 2nd-instar caterpillars	[152,165]
Peltophorum dubium (Spreng.) Taub.	S	TI	Survival = 44.3% at 1.6%	*Anagasta kuehniella*	Artificial diet	4th-stage larvae	[166]
Pterodon emarginatus Vogel	F	EO	LC50 = 371.6 µg/mL	*Aedes aegypti*	Mortality determination	4th-stage larvae	[167]
**Lamiaceae**							
Hyptis crenata Pohl ex Benth.	AP	EO	PE = 94.4% at 2.5% concentration	*Rhipicephalus microplus*	Adult immersion test	Engorged females	[79]
**Lauraceae**							

Ocotea lancifolia (Schott) Mez	L	Eth	PE = 34.50% at 0.2 % concentration	*Rhipicephalus microplus*	Adult immersion test	Engorged females	[168]
Ocotea velloziana (Meisn.) Mez	SW	Eth	LC50 = 30.2 and 213.70 µg/mL	*Aedes aegypti*	Mortality determination and bioassay-directed fractionation	larvae	[161]
**Loganiaceae**							
Strychnos pseudoquina A. St.-Hil.	S	Eth	PE = 48.03% at 0.2 % concentration	*Rhipicephalus microplus*	Adult immersion test	Engorged females	[168]
**Melastomataceae**							
Mouriri elliptica Mart.	L	EA and M	12.43- 20 µg/mL	Generic α-amylase and generic acetylcholinesterase	*In vitro* inhibition assay	Purified enzyme	[169]
**Meliaceae**							
Cabralea canjerana (Vell.) Mart.	SE	EA	330 mg/kg	*Spodoptera frugiperda*	Artificial diet	3rd instar larvae	[170]
Guarea guidonia (L.) Sleumer	R	HE	50 µg	*Rhodnius milesi*	Topical test	4th-instar nymphs	[171]
Guarea kunthiana A. Juss.	S and F	Eth	LC50 = 14.44 and 169.93 µg/mL	*Aedes aegypti* and *Rhipicephalus microplus*	Mortality determination and adult immersion test	3rd-stage larvae andengorged females	[168,172,173]
**Myrtaceae**							
Eugenia involucrata DC.	L	EO	LC50 = 199.3 µg/mL	*Aedes aegypti*	Mortality determination	3rd-stage larvae	[169]
Psidium laruotteanum Cambess.	L	Eth	2% concentration	*Diabrotica speciosa*	soil treatment	3-day old larvae	[174]
**Piperaceae**							
Piper aduncum L.	L	EO	LC50 = 289.9 ppm	*Aedes aegypti*	Mortality determination	Larvae	[175]
**Rubiaceae**							
Psychotria capitata Ruiz & Pav.	S	Eth	Efficacy = 71.05%	Sitophilus zeamais	Artificial diet	nonsexed 10-day-old adults	[176]
Psychotria deflexa DC. subsp. deflexa	S	Eth	Efficacy = 95.83%	*Spodoptera frugiperda*	Artificial diet	1-day-old caterpillar	[176]
Psychotria hoffmannseggiana	S	Eth	Efficacy = 56.00%	Sitophilus zeamais	Artificial diet	nonsexed 10-day-old adults	[176]

(Willd. ex Schult.) Müll.Arg.							
Psychotria prunifolia (Kunth) Steyerm.	L	Eth	LT50 = 89.73 h and Efficacy = 60.21%	*Sitophilus zeamais* and *Sitotroga cerealella*	Artificial diet and residual contact	90-day-old unsexed adults and larvae	[177,178]
**Rutaceae**							
Zanthoxylum rhoifolium Lam.	F	EO	Efficiency = 98.3% at 2%	*Bemisia tabaci*	No-choice and free-choice bioassay	adults whiteflies	[179]
Zanthoxylum riedelianum Engl.	F	EO	Efficiency = 94.2% at 1.5%	*Bemisia tabaci*	No-choice and free-choice bioassay	adults whiteflies	[179]
**Salicaceae**							
Casearia sylvestris Sw. var. sylvestris	S	HE	LC50 = 232.4 µg/mL	*Aedes aegypti*	Mortality determination	3rd-stage larvae	[153]
**Sapindaceae**							
Serjania lethalis A.St.-Hil.	R	Eth	LC50 = 285.76 µg/mL	*Aedes aegypti*	Mortality determination	3rd-stage larvae	[153]
**Simaroubaceae**							
Simarouba versicolor A.St.-Hil.	RB	Eth	50 µg	*Rhodnius milesi*	Topical test	4th-instar nymphs	[171]
**Smilacaceae**							
Smilax brasiliensis Spreng.	L	CHE	LC50 = 469.78 µg/mL	*Culex quinquefasciatus*	Mortality determination	Larvae	[180]

Part of plant[1] (Aerial parts=AP; Fruits= F; Flowers= F; Nuts=N; Leaves= L; Root bark= RB; Root= R; Root Wood= RW; Stem= S; Stem Bark= SB; Bark= B; Send= SE; Heartwood=HW).
Type of extract[2] (Ethanolic= Eth; Ethyl-acetate= EA; Methanol-acetate= MA; Hexane= HE; Chloroform= Chl; Essential oils= EO;Methanol= M; Hydroethanolic= HEth; Cyclohexane= CHE; Trypsin inhibitor=TI).

Table S9 Molluscicidal activity from different species of Cerrado. Information about part from plant and extract preparation, dose tested, model and method analyzed.

Family/Species	Part[1]	Extract[2]	Dose /Concentration	Activity against	Technique	Experimental model	Reference
**Caryocaraceae**							
Caryocar brasiliense Cambess.	L or B	Eth	100 ppm = 90% mortality in 48 h	*Biomphalaria glabrata*	Snails submersion for 24 h	*Biomphalaria glabrata*	[181]
**Dilleniaceae**							
Davilla rugosa Poir.	L	DM	LC100/72 h = 100 ppm	*Biomphalaria glabrata*	Snails submersion for 24 h	*Biomphalaria glabrata*	[82]
**Fabaceae**							
Stryphnodendron adstringens (Mart.) Coville	L or B	Eth	50 ppm = 60-90% mortality in 48 h	*Biomphalaria glabrata*	Snails submersion for 24 h	*Biomphalaria glabrata*	[181]
Stryphnodendron polyphyllum Mart.	L or B	Eth	50 ppm = 70% mortality in 48 h	*Biomphalaria glabrata*	Snails submersion for 24 h	*Biomphalaria glabrata*	[181]
Hymenaea stigonocarpa Mart. ex Hayne	B or L	DM or M	40 < LC100/72 h > 100 ppm	*Biomphalaria glabrata*	Snails submersion for 24 h	*Biomphalaria glabrata*	[82]
Plathymenia reticulata Benth.	L	M	LC100/72 h = 100 ppm	*Biomphalaria glabrata*	Snails submersion for 24 h	*Biomphalaria glabrata*	[82]
**Malpighiaceae**							
Byrsonima coccolobifolia Kunth	L	DM	LC100/72 h = 100 ppm	*Biomphalaria glabrata*	Snails submersion for 24 h	*Biomphalaria glabrata*	[82]
Byrsonima intermedia A.Juss.	L	M	LC100/72 h = 20 ppm	*Biomphalaria glabrata*	Snails submersion for 24 h	*Biomphalaria glabrata*	[82]
Byrsonima verbascifolia (L.) DC.	L or B	DM or M or Aq	40 < LC100/72 h > 60 ppm	*Biomphalaria glabrata*	Snails submersion for 24 h	*Biomphalaria glabrata*	[82]
**Metteniusaceae**							
Emmotum nitens (Benth.) Miers	S	M	LC100/72 h = 100 ppm	*Biomphalaria glabrata*	Snails submersion for 24 h	*Biomphalaria glabrata*	[82]
**Myrtaceae**							
Eugenia dysenterica (Mart.) DC.	L	DM and Eth	LC100/72 h = 100 ppm and 100 ppm = 100% mortality in 48 h	*Biomphalaria glabrata*	Snails submersion for 24 h	*Biomphalaria glabrata*	[82,181]
**Styracaceae**							
Styrax camporum Pohl	L	DM	LC100/72 h = 100 ppm	*Biomphalaria glabrata*	Snails submersion for 24 h	*Biomphalaria glabrata*	[82]

**Vochysiaceae**							
Qualea parviflora Mart.	B	Aq	LC100/72 h = 100 ppm	*Biomphalaria glabrata*	Snails submersion for 24 h	*Biomphalaria glabrata*	[82]

Part of plant[1] (Leaves= L; Stem= S; Bark= B).
Type of extract[2] (Aqueous= Aq; Dichloromethane= DM; Ethanolic= Eth; Methanol= M).

Table S10 Secondary metabolites isolated from Cerrado plants that present toxic activity. Information on chemical class, tested dose, model and analyzed method.

Identified compound	Species	Class	Concentration	Technique	Experimental model	Reference
**Cytotoxic**						
Octacosane **(1)**	*Pyrostegia venusta* (Ker Gawl.) Miers	Alkanes	41.08 μg/mL	MTT assay	B16F10-Nex2 cells	[9]
Triacontane **(2)**	*Pyrostegia venusta* (Ker Gawl.) Miers	Alkanes	20.9 μg/mL	MTT assay	B16F10-Nex2 cells	[9]
Sellovicine B **(3)**	*Croton velutinus* Baill.	Phenylpropanoids	6.8-18.3 μM/mL	Alamar Blue assay	HepG2 and MCF-7 cells	[182]
(E)-1-(7,8-epoxypropen) phenyl benzoate **(4)**	*Croton velutinus* Baill.	Phenylpropanoids	11.1-19.4 μm/mL	Alamar Blue assay	HepG2 and MCF-7 cells	[182]
Phloretin **(5)**	*Lippia salviaefolia* Cham.	Flavonoids	20-40 μM	MTT assay	HEK-293 and M14 cells	[44]
[1-9-NαC]-crourorb A1 (6)	*Croton urucurana* Baill.	Corbitides	50- 200 μg/mL	WST-1 assay	Huh-7 cells	[23]
d-tocotrienol **(7)**	*Kielmeyera coriácea* Mart. & Zucc.	Terpenes	5 and 10 μg/mL	Trypan blue assay	MDA-MB-435, HCT-8, HL-60 and SF-295 cells	[12]
d-tocotrienol peroxy-dimer **(8)**	*Kielmeyera coriacea* Mart. & Zucc.	Terpenes	5 and 10 μg/mL	Trypan blue assay	MDA-MB-435, HCT-8, HL-60 and SF-295 cells	[12]
11β-hydroxypristimerin **(9)**	*Salacia crassifólia* (Mart. ex Schult.) G.Don	Terpenes	8.7 μM	Formazan Endpoint assay	NCI-60 and 786-0 cells	[183]
Pristimerin **(10)**	*Salacia crassifolia* (Mart. ex Schult.) G.Don	Terpenes	0.3-1.2 μM	Formazan Endpoint assay	NCI-60, UO-31, T-47D and A549 cells	[183]
Zornioside (C-glycosylated dihydrochalcone) **(11)**	*Zornia brasiliensis* Vogel	Flavonoids	37.26 μM	MTT assay	HL60 cells	[184]
Vallesiachotamine **(12)**	*Palicourea rigida* Kunth	Alkaloids	50 μM	MTT assay	SK-MEL-37 cells	[185]
β-friedelinol **(13)**	*Cheiloclinium cognatum* (Miers) A.C.Sm.	Terpenes	15.75-55.20 μmol/L-1	MTT assay	THP-1, K562 and PMBC cells	[16]
α-amyrin **(14)**	*Cheiloclinium cognatum* (Miers) A.C.Sm.	Terpenes	9.92-55.65 μmol/L-1	MTT assay	THP-1, K562 and PMBC cells	[16]
Dioscin **(15)**	*Chamaecostus subsessilis* (Nees & Mart.) C.D.Specht & D.W.Stev.	Saponins	4.6 and 15.1 μg/mL	MTT assay	HL60 cells	[19]
Gracillin **(16)**	*Chamaecostus subsessilis* (Nees & Mart.) C.D.Specht & D.W.Stev.	Saponins	4.6 and 15.1 μg/mL	MTT assay	HL60 cells	[19]
**Mutagenic**						
Isoobtusilactone A **(17)**	*Aiouea trinervis* Meisn.	Butanolides	0.1- 0.3 mg/mL	SMART test	*Drosophila melanogaster*	[186]
Obtusilactone A **(17)**	*Aiouea trinervis* Meisn.	Butanolides	0.1- 0.3 mg/mL	SMART test	*Drosophila melanogaster*	[186]

Amentoflavone **(18)**	*Byrsonima crassa* Nied.	Flavonoids	0.1- 0.3 mg/mL	Ames test	*Salmonella typhimurium*	[50]
**Antibacterial**						
Gallic acid **(19)**	*Cochlospermum regium* (Mart. ex Schrank) Pilg.	Tannines	MIC = 50.8 - 101.6 μg/mL	Broth Microdilution Assay	*Staphylococcus aureus*, *Staphylococcus epidermidis*, *Escherichia coli*, *Pseudomonas aeruginosa* and E*nterococcus faecalis*	[187]
Tannic acid **(20)**	*Cochlospermum regium* (Mart. ex Schrank) Pilg.	Tannines	MIC = 250-500 μg/mL	Broth Microdilution Assay	*Staphylococcus aureus* (29213)and *Escherichia coli* (25922) strains	[71]
Spathulenol **(21)**	*Psidium guineense* Sw.	Terpenes	MIC = 31.25-125 μg/mL	Broth Microdilution Assay	*Klebsiella pneumoniae*, P*seudomonas aeruginosa* and *Acinetobacter baumannii*	[95]
Mauritic acid **(22)**	*Mauritia flexuosa* L.f.	Terpenes	MIC = 231.9 μg/mL	REMA method	*Mycobacterium tuberculosis*	[187]
5-(3''-hydroxypropyl)- 7- methoxy-2-(3',4' - dimethoxyphenyl) benzofuran **(23)**	*Styrax ferrugineus* Nees & Mart.	Arylpropanoids	MIC= 10-20 μg/mL	Broth Microdilution Assay	*Staphylococcus aureus*	[188]
5-(3''-hydroxypropyl)-7-methoxy-2-(3',4'-methylenedioxyphenyl) benzofuran **(24)**	*Styrax ferrugineus* Nees & Mart.	Arylpropanoids	MIC= 10-20 μg/mL	Broth Microdilution Assay	*Staphylococcus aureus*	[188]
5- [3''-(b-d-glucopyranosyloxy)propyl]-7-methoxy-2-(3' ,4' - methylenedioxyphenyl) benzofuran **(25)**	*Styrax ferrugineus* Nees & Mart.	Arylpropanoids	MIC= 10-20 μg/mL	Broth Microdilution Assay	*Staphylococcus aureus*	[188]
5-[3''-(b-d-glucopyranosyloxy)propyl]-7-methoxy-2-(3' ,4' - dimethoxyphenyl) benzofuran **(26)**	*Styrax ferrugineus* Nees & Mart.	Arylpropanoids	MIC= 10-20 μg/mL	Broth Microdilution Assay	*Staphylococcus aureus*	[188]
Dihydrodehydrodiconiferyl alcohol **(27)**	*Styrax ferrugineus* Nees & Mart.	Arylpropanoids	MIC= 10-20 μg/mL	Broth Microdilution Assay	*Staphylococcus aureus*	[188]
**Antifungal**						
Gallic acid **(19)**	*Cochlospermum regium* (Mart. ex Schrank) Pilg.	Tannines	MIC = 31.25 μg/mL	Broth Microdilution Assay	*Candida albicans*	[71]

Tannic acid **(20)**	*Cochlospermum regium* (Mart. ex Schrank) Pilg.	Tannines	MIC = 125-250 µg/mL	Broth Microdilution Assay	*Candida albicans*, *Candida krusei* and *Candida glabrata*	[71]
Mauritic acid **(22)**	*Mauritia flexuosa* L.f.	Terpenes	MIC = 203.5 µg/mL	Broth Microdilution Assay	*Candida albicans*	[187]
5-(3''-hydroxypropyl)- 7- methoxy-2-(3',4' -dimethoxyphenyl) benzofuran (23)	*Styrax ferrugineus* Nees & Mart.	Arylpropanoids	MIC= 10-20 µg/mL	Broth Microdilution Assay	*Staphylococcus aureus*	[188]
5-(3''-hydroxypropyl)-7-methoxy-2-(3',4'-methylenedioxyphenyl) benzofuran (24)	*Styrax ferrugineus* Nees & Mart.	Arylpropanoids	MIC= 10-20 µg/mL	Broth Microdilution Assay	*Candida sphaerospermum* and *Candida albicans*	[188]
5- [3''-(b-d-glucopyranosyloxy)propyl]-7-methoxy-2-(3' ,4' - methylenedioxyphenyl) benzofuran (25)	*Styrax ferrugineus* Nees & Mart. Nees & Mart.	Arylpropanoids	MIC= 10-20 µg/mL	Broth Microdilution Assay	*Candida sphaerospermum* and *Candida albicans*	[188]
5-[3''-(b-d-glucopyranosyloxy)propyl]-7-methoxy-2-(3' ,4' - dimethoxyphenyl) benzofuran (26)	*Styrax ferrugineus* Nees & Mart.	Arylpropanoids	MIC= 10-20 µg/mL	Broth Microdilution Assay	*Candida sphaerospermum* and *Candida albicans*	[188]
Dihydrodehydrodiconiferyl alcohol **(27)**	*Styrax ferrugineus* Nees & Mart.	Arylpropanoids	MIC= 10-20 µg/mL	Broth Microdilution Assay	*Candida sphaerospermum* and *Candida albicans*	[188]
Vatacarpan **(28)**	*Vatairea macrocarpa* (Benth.) Ducke	Flavonoids	MIC = 0.98 µg/mL	Broth Microdilution Assay	*Candida albicans*	[111]
Brachydin B (BR-B) **(29)**	*Arrabidaea brachypoda* (DC.) Bureau	Flavonoids	MIC = 161 µg/mL	Broth Microdilution Assay	*Candida albicans*	[189]
4'-O-Methyl-catechin **(30)**	*Curatella americana* L.	Catechins	MIC = 31.3 -125 µg/mL	Broth Microdilution Assay	*Candida albicans*, *Candida tropicalis* and *Candida parapsilosis*	[110]
Epicatechin-3-O-gallate **(31)**	*Curatella americana* L.	Catechins	MIC = 31.3 -125 µg/mL	Broth Microdilution Assay	*Candida albicans*, *Candida tropicalis* and *Candida parapsilosis*	[110]
Isoverbascoside **(32)**	*Pyrostegia venusta* (Ker Gawl.) Miers	Phenylpropanoids	MIC = 0.7-6.0 µg/mL	Broth Microdilution Assay	*Candida albicans*, *Candida krusei*, *Candida tropicalis*, *Candida parapsilosis* and *Candida guilhermondii*	[190]

Verbascoside **(33)**	*Pyrostegia venusta* (Ker Gawl.) Miers *Pyrostegia venusta* (Ker Gawl.) Miers	Phenylpropanoids	MIC = 0.7-1.5 µg/mL	Broth Microdilution Assay	*Candida albicans, Candida krusei, Candida tropicalis, Candida parapsilosis and Candida guilhermondii*	[190]
Rapanone **(34)**	*Connarus suberosus* Planch.	Quinones	MIC = 15.62 µg/mL	Broth Microdilution Assay	*Candida albicans*	[121]
Suberonone **(35)**	*Connarus suberosus* Planch.	Quinones	MIC = 125-250 µg/mL	Broth Microdilution Assay	*Candida parapsilosis* and *Candida glabrata*	[121]
(2S)- and (2R)-30 ,40,5,6-Tetrahydroxyflavanone-7-O-b-glucopyranoside **(36)**	*Lippia origanoides* Kunth	Flavonoids	MIC = 5-12 µg/mL	Broth Microdilution Assay	*Candida albicans* and *Cladosporium cladosporioides*	[188]
(2S)- and (2R)-30 ,40 ,5,8-tetrahydroxyflavanone-7-O-b-glucopyranoside **(37)**	*Lippia origanoides* Kunth	Flavonoids	MIC = 31.2- 62.5 µg/mL	Broth Microdilution Assay	*Candida krusei*, *Candida parapsilosis* and *Cryptococcus neoformans*	[191]
Biochanin A **(38)**	*Lippia lupulina* Cham.	Flavonoids	MIC = 62.5 µg/mL	Broth Microdilution Assay	*Candida krusei* and *Cryptococcus neoformans*	[191]
**Antiparasitic**						
Sellovicin B **(3)**	*Croton velutinus* Baill.	Phenylpropanoids	EC50 = 9 µM	Parasite Integrity and Motility	Trypomastigotes	[182]
(E)-1-(7,8-epoxypropen) phenyl benzoate **(4)**	*Croton velutinus* Baill.	Phenylpropanoids	EC50 = 9.58 µM	Parasite Integrity and Motility	Trypomastigotes	[182]
gallic acid **(19)**	*Stryphnodendron obovatum* Benth.	Tannines	IC50 = 1.7 µg/mL^{-1}	MTT assay	Promastigotes	[141]
Cupacinoside **(39)**	*Cupania cinérea* Poepp. & Endl.	Terpenes	IC50 = 1.3 µM	Tritiated Hypoxanthine Incorporation assay	K1 strain	[192]
6′-de-O-acetylcupacinoside **(40)**	*Cupania cinérea* Poepp. & Endl.	Terpenes	IC50 = 1.8 µM	Tritiated Hypoxanthine Incorporation assay	K1 strain	[192]
Cupacinoxepin **(41)**	*Cupania cinerea* Poepp. & Endl.	Terpenes	IC50 = 8.7 µM	Tritiated Hypoxanthine	K1 strain	[192]

				Incorporation assay		
Casearin A **(42)**	*Casearia sylvestris* Sw.	Terpenes	4.45-9.48 μg/mL^{-1}	MTT assay	Promastigotes	[146]
Casearin B **(43)**	*Casearia sylvestris* Sw.	Terpenes	0.53- 2.77 μg/mL^{-1}	MTT assay	Trypomastigotes	[146]
Casearin J **(44)**	*Casearia sylvestris* Sw.	Terpenes	4.45 -9.48 μg/mL^{-1}	MTT assay	Promastigotes	[146]
Casearin G **(45)**	*Casearia sylvestris* Sw.	Terpenes	0.53- 2.77 μg/mL^{-1}	MTT assay	Trypomastigotes	[146]
Tingenone **(46)**	*Cheiloclinium cognatum* (Miers) A.C.Sm.	Terpenes	IC50 = 486.6 μM	N/S	Trypomastigotes	[193]
Tingenol **(47)**	*Cheiloclinium cognatum* (Miers) A.C.Sm.	Terpenes	IC50 = 306.9 μM	N/S	Trypomastigotes	[193]
Solamargine **(48)**	*Solanum lycocarpum* A.St.-Hil.	Alkaloids	IC50 = 3.0 and 8.1 μg/mL^{-1}	Flagellar Motility Avaliation	Promastigotes	[194]
Solamargine **(48)**	*Solanum palinacanthum* Dunal	Alkaloids	IC50 = 15.3 μg/mL^{-1}	MTT assay	Epimastigotes	[148]
ent-kaurane **(49)**	*Aldama discolor* (Baker) E.E.Schill. & Panero	Terpenes	IC50 = 2.5 μM	Tritiated Hypoxanthine Incorporation assay	K1 strain	[195]
ent-pimarane **(50)**	*Aldama discolor* (Baker) E.E.Schill. & Panero	Terpenes	IC50 = 3.8 μM	Tritiated Hypoxanthine Incorporation assay	K1 strain (resistant to chloroquine and pyrimethamine)	[195]
Epigallocatechin gallate **(51)**	*Stryphnodendron obovatum* Benth.	Flavonoids	IC50 = 16.3 μg/mL^{-1}	MTT assay	Promastigotes	[141]
Solasodine **(52)**	*Solanum lycocarpum* Benth.	Alkaloids	IC50 = 4.7 and 10.8 μg/mL^{-1}	Flagellar Motility Avaliation	Promastigotes	[194]
Solasonine **(53)**	*Solanum lycocarpum* Benth.	Alkaloids	IC50 = 4.7 and 22.7 μg/mL^{-1}	Flagellar Motility Avaliation	Promastigotes	[194]
**Insecticidal**						
(+)-dicentrine **(54)**	*Ocotea velloziana* (Meisn.) Mez	Alkaloids	LC50 = 30.2 μg/mL^{-1}	Bioassay-Directed Fractionation	larvae	[161]
Alepterolic acid **(55)**	*Copaifera multijuga* Hayne	Terpenes	LC50 = 0.7 ppm	Mortality Determination	larvae	[164]
Maackiain **(56)**	*Bowdichia virgilioides* Kunth	Flavonoids	LC50 = 21.95 μg/mL^{-1}	Mortality Determination	4th-stage larvae	[163]
Melianodiol **(57)**	*Guarea kunthiana* A.Juss.	Protolimonoids	LC50 = 14.44 μg/mL^{-1}	Mortality Determination	3rd-stage larvae	[196]

3β-O-tigloylmelianol **(58)**	*Guarea kunthiana A.Juss.*	Protolimonoids	1%	Adult Immersion test	engorged females	[173]
Astilbin **(59)**	*Dimorphandra mollis* Benth.	Flavonoids	10- 20 mg/mL^{-1}	Topical Application and Artificial Diet	eggs neonate larvae	[197–199]
Tectoquinone **(60)**	*Connarus suberosus* Planch.	Quinones	LC50 1.1 μg/mL	Mortality Determination	larvae	[200]
Lectin (N/S)	*Myracrodruon urundeuva* M. Allemão	(N/A)	LC50 = 202 μg/mL^{-1}	Mortality Determination	4th-stage larvae	[201]

Not Specificid=N/S

Cells lines: MDA-MB-435 (melanoma), HCT-8 (colon), HL-60 (leukemia), SF-295 (glioblastoma), B16F10-Nex2 (murine melanoma), 786-0 (kidney adenocarcinoma),Huh-7 (human hepatocarcinoma), HepG2 (hepatocyte carcinoma), HEK-293 (Human embryonic kidney), M14 (melanoma), NCI-60 (Mammalian Cell Lines), UO31 (Human Kidney Renal Cell Carcinoma), A549 (adenocarcinomic human), SK-MEL-37 (Human Melanoma), THP-1 (acute monocytic leukemia ATCCTIB-202), K562 (chronic myeloid leukemia ATCC CRL-3343) and PBMCs (Peripheral Blood Mononuclear).

References

1. Mesquita, M.L. de; Paula, J.E. de; Pessoa, C.; Moraes, M.O. de; Costa-Lotufo, L.V.; Grougnet, R.; Michel, S.; Tillequin, F.; Espindola, L.S. Cytotoxic activity of Brazilian Cerrado plants used in traditional medicine against cancer cell lines. *Journal of Ethnopharmacology* **2009**, *123*, 439–445, doi:10.1016/j.jep.2009.03.018.
2. Prado, L.G.; Arruda, H.S.; Peixoto Araujo, N.M.; de Oliveira Braga, L.E.; Banzato, T.P.; Pereira, G.A.; Figueiredo, M.C.; Ruiz, A.L.T.G.; Eberlin, M.N.; de Carvalho, J.E.; et al. Antioxidant, antiproliferative and healing properties of araticum (Annona crassiflora Mart.) peel and seed. *Food Research International* **2020**, *133*, 109168, doi:10.1016/j.foodres.2020.109168.
3. Munari, C.C.; Alves, J.M.; Bastos, J.K.; Tavaresa, D.C. Evaluation of the genotoxic and antigenotoxic potential of *Baccharis dracunculifolia* extract on V79 cells by the comet assay. *Journal of Applied Toxicology* **2010**, *30*, 22–28, doi:10.1002/jat.1467.
4. de Moura, D.F.; Rocha, T.A.; Barros, D. de M.; da Silva, M.M.; de Lira, M.A. da C.; dos Santos Souza, T.G.; da Silva, C.J.A.; de Aguiar Júnior, F.C.A.; Chagas, C.A.; da Silva Santos, N.P.; et al. Evaluation of the cytotoxicity, oral toxicity, genotoxicity, and mutagenicity of the latex extracted from *Himatanthus drasticus* (Mart.) Plumel (Apocynaceae). *Journal of Ethnopharmacology* **2020**, *253*, doi:10.1016/j.jep.2020.112567.
5. Almeida, L.M.; Prado, A.D.L.; Xavier-Silva, K.R.; Firmino, M.T.; Paula, M.I.M.; Gomes, P.N.; Paula, J.A.M.; Bailão, E.F.L.C. Cytotoxic effect of *Vernonanthura polyanthes* leaves aqueous extracts. *Brazilian journal of biology = Revista brasleira de biologia* **2020**, doi:10.1590/1519-6984.225281.
6. Rocha, J.D.; da Silva Ferreira, J.; Vieira Silva, J.G.; Silva Fernandes, A.; Hollanda Véras, J.; Madureira de Almeida, L.; Magalhães Teles, A.; Luiz Borges, L.; Chen-Chen, L.; Luiz Cardoso Bailão, E.F. In vitro hematotoxicity of *Vernonanthura polyanthes* leaf aqueous extract and its fractions. *Drug and Chemical Toxicology* **2020**, *0*, 1–9, doi:10.1080/01480545.2020.1802481.
7. da Silva, A.C.N.; do Nascimento, R.M.C.; Rodrigues, D.C. do N.; Ferreira, P.M.P.; Pessoa, C.; Lima, D.J.B.; Moraes Filho, M.O. de; de Almeida, R.M.; Ferreira, S.R.; Fujiwara, R.T.; et al. In vitro activity evaluation of seven Brazilian Asteraceae against cancer cells and *Leishmania amazonensis*. *South African Journal of Botany* **2019**, *121*, 267–273, doi:10.1016/j.sajb.2018.11.008.
8. Bedir, E.; Pereira, A.M.S.; Khan, S.I.; Chittiboyina, A.; Moraes, R.M.; Khan, I.A. A New β-lapachone derivative from *Distictella elongata* (vahl) urb. *Journal of the Brazilian Chemical Society* **2009**, *20*, 383–386, doi:10.1590/S0103-50532009000200026.
9. Figueiredo, C.R.; Matsuo, A.L.; Pereira, F. V; Rabaça, A.N.; Farias, C.F.; Girola, N.; Massaoka, M.H.; Azevedo, R.A.; Scutti, J.A.B.; Arruda, D.C.; et al. *Pyrostegia venusta* heptane extract containing saturated aliphatic hydrocarbons induces apoptosis on B16F10-Nex2 melanoma cells and displays antitumor activity in vivo. *Pharmacognosy magazine* **2014**, *10*, S363-76, doi:10.4103/0973-1296.133284.
10. Andrade, L.S.; Santos, D.B.; Castro, D.B.; Guillo, L.A.; Chen-Chen, L. Absence of antimutagenicity of *Cochlospermum regium* (Mart. and Schr.) Pilger 1924 by micronucleus test in mice. *Brazilian Journal of Biology* **2008**, *68*, 155–159, doi:10.1590/S1519-69842008000100022.
11. Figueiredo, C.R.; Matsuo, A.L.; Massaoka, M.H.; Girola, N.; Azevedo, R.A.; Rabaça, A.N.; Farias, C.F.; Pereira, F.V.; Matias, N.S.; Silva, L.P.; et al. Antitumor activity of *Kielmeyera coriacea* leaf constituents in experimental melanoma, tested in vitro and in vivo in syngeneic mice. *Advanced Pharmaceutical Bulletin* **2014**, *4*, 429–436, doi:10.5681/apb.2014.063.
12. De Mesquita, M.L.; Araújo, R.M.; Bezerra, D.P.; Filho, R.B.; De Paula, J.E.; Silveira, E.R.; Pessoa, C.; De Moraes, M.O.; Costa Lotufo, L.V.; Espindola, L.S. Cytotoxicity of δ-tocotrienols from *Kielmeyera coriacea* against cancer cell lines. *Bioorganic and Medicinal Chemistry* **2011**, *19*, 623–630, doi:10.1016/j.bmc.2010.10.044.
13. Ombredane, A.S.; Araujo, V.H.S.; Borges, C.O.; Costa, P.L.; Landim, M.G.; Pinheiro, A.C.; Szlachetka, Í.O.; Benedito, L.E.C.; Espindola, L.S.; Dias, D.J.S.; et al. Nanoemulsion-based systems as a promising approach for enhancing the antitumoral activity of pequi oil (*Caryocar brasilense* Cambess.) in breast cancer cells. *Journal of Drug Delivery Science and Technology* **2020**, *58*, 101819, doi:10.1016/j.jddst.2020.101819.
14. Espindola, L.S.; Dusi, R.G.; Demarque, D.P.; Braz-Filho, R.; Yan, P.; Bokesch, H.R.; Gustafson, K.R.; Beutler, J.A. Cytotoxic triterpenes from *Salacia crassifolia* and metabolite profiling of celastraceae species. *Molecules* **2018**, *23*, 1–11, doi:10.3390/molecules23061494.
15. Carneiro, C.C.; Silva, C.R.; Menezes, A.C.S.; Pérez, C.N.; Chen-Chen, L. Assessment of genotoxic, cytotoxic, and protective effects of *Salacia crassifolia* (Mart. Ex. Schult.) G. Don. stem bark fractions in mice. *Genetics and Molecular Research* **2013**, *12*, 2167–2177, doi:10.4238/2013.July.3.1.
16. Gonçalves Pereira, R.C.; Gontijo Evangelista, F.C.; dos Santos Júnior, V.S.; de Paula Sabino, A.; Gonçalves Maltarollo, V.; de Freitas, R.P.; Pains Duarte, L. Cytotoxic Activity of Triterpenoids from *Cheiloclinium cognatum* Branches against Chronic and Acute Leukemia Cell Lines. *Chemistry and Biodiversity* **2020**, *17*, doi:10.1002/cbdv.202000773.
17. da Cunha Demenciano, S.; Lima e Silva, M.C.B.; Farias Alexandrino, C.A.; Kato, W.H.; de Oliveira Figueiredo, P.; Garcez, W.S.; Campos, R.P.; de Cássia Avellaneda Guimarães, R.; Sarmento, U.C.; Bogo, D. Antiproliferative activity and antioxidant potential of extracts of *Garcinia gardneriana*. *Molecules* **2020**, *25*, 1–20, doi:10.3390/molecules25143201.
18. De Sousa, W.C.; Paz, A.T.S.; Rocha, J.D.; Da Conceição, E.C.; De Almeida, L.M.; Chen, L.C.; Borges, L.L.; Bailão, E.F.L.C. In vivo assessment of cyto/genotoxic, antigenotoxic and antifungal potential of *Costus spiralis* (Jacq.) Roscoe leaves and stems. *Anais da Academia Brasileira de Ciencias* **2018**, *90*, 1565–1577, doi:10.1590/0001-3765201720170714.

19. De Siqueira, E.P.; Braga, A.C.S.; Galligani, F.; De Souza-Fagundes, E.M.; Cota, B.B. Saponins from the rhizomes of *Chamaecostus* subsessilis and their cytotoxic activity against HL60 human promyelocytic leukemia cells. *Journal of Pharmacy and Pharmacognosy Research* **2020**, *8*, 466–474.
20. Elias, S.T.; Borges, G.A.; Amorim, D.A.; Rêgo, D.F.; Simeoni, L.A.; Silveira, D.; Fonseca-Bazzo, Y.M.; Paula, J.E.; Fagg, C.W.; Barros, I.M.C.; et al. Radiation induced a supra-additive cytotoxic effect in head and neck carcinoma cell lines when combined with plant extracts from Brazilian Cerrado biome. *Clinical Oral Investigations* **2014**, *19*, 637–646, doi:10.1007/s00784-014-1289-z.
21. Elias, S.T.; Macedo, C.C.S.; Simeoni, L.A.; Silveira, D.; Magalhães, P.O.; Lofrano-Porto, A.; Coletta, R.D.; Neves, F.A.R.; Guerra, E.N.S. Cytotoxic effect of *Erythroxylum daphnite*s extract is associated with G1 cell cycle arrest and apoptosis in oral squamous cell carcinoma. *Cell Cycle* **2016**, *15*, 948–956, doi:10.1080/15384101.2016.1151583.
22. Abreu, L.S.; do Nascimento, Y.M.; do Espirito-Santo, R.F.; Meira, C.S.; Santos, I.P.; Brandão, R.B.; Souto, A.L.; Guedes, M.L.S.; Soares, M.B.P.; Villarreal, C.F.; et al. Phenylpropanoids from *Croton velutinus* with cytotoxic, trypanocidal and anti-inflammatory activities. *Fitoterapia* **2020**, *145*, 104632, doi:10.1016/j.fitote.2020.104632.
23. de Matos Cândido-Bacani, P.; Ezan, F.; de Oliveira Figueiredo, P.; Matos, M. de F.C.; Rodrigues Garcez, F.; Silva Garcez, W.; Baffet, G. [1–9-NαC]-crourorb A1 isolated from *Croton urucurana* latex induces G2/M cell cycle arrest and apoptosis in human hepatocarcinoma cells. *Toxicology Letters* **2017**, *273*, 44–54, doi:10.1016/j.toxlet.2017.03.020.
24. Cândido-Bacani, P.D.M.; Figueiredo, P.D.O.; Matos, M.D.F.C.; Garcez, F.R.; Garcez, W.S. Cytotoxic Orbitide from the Latex of *Croton urucurana*. *Journal of Natural Products* **2015**, *78*, 2754–2760, doi:10.1021/acs.jnatprod.5b00724.
25. Santos, M.G.; Almeida, V.G.; Avelar-Freitas, B.A.; Grael, C.F.F.; Gregório, L.E.; Pereira, W.F.; Brito-Melo, G.E.A. Phytochemical screening of the dichloromethane–ethanolic extract of E*riosema campestre* var. *macrophylum* roots and its antiproliferative effect on human peripheral blood lymphocytes. *Revista Brasileira de Farmacognosia* **2016**, *26*, 464–470, doi:10.1016/j.bjp.2015.08.009.
26. Baldivia, D. da S.; Leite, D.F.; de Castro, D.T.H.; Campos, J.F.; Dos Santos, U.P.; Paredes-Gamero, E.J.; Carollo, C.A.; Silva, D.B.; Souza, K. de P.; Dos Santos, E.L. Evaluation of in vitro antioxidant and anticancer properties of the aqueous extract from the stem bark of *Stryphnodendron adstringens*. *International Journal of Molecular Sciences* **2018**, *19*, doi:10.3390/ijms19082432.
27. Vilar, J.B.; D'Oliveira, M.I.P.; Santos, S. da C.; Chen, L.C. Cytotoxic and genotoxic investigation on barbatimão [*Stryphnodendron adstringens* (Mart.) Coville, 1910] extract. *Brazilian Journal of Pharmaceutical Sciences* **2010**, *46*, 687–694, doi:10.1590/S1984-82502010000400010.
28. Oliveira-Alves, S.C.; Pereira, R.S.; Pereira, A.B.; Ferreira, A.; Mecha, E.; Silva, A.B.; Serra, A.T.; Bronze, M.R. Identification of functional compounds in baru (*Dipteryx alata* Vog.) nuts: Nutritional value, volatile and phenolic composition, antioxidant activity and antiproliferative effect. *Food Research International* **2020**, *131*, doi:10.1016/j.foodres.2020.109026.
29. Santana, G.M.; Deus, M.S.M.; Sousa, J.M.C.; Ferreira, P.M.P.; Fernandes, H.B.; Peron, A.P. Ação antimitótica e antimutagênica do ritidoma de *Hymenaea stigonocarpa* Mart ex Hayne na divisão celular. *Brazilian Journal of Biology* **2016**, *76*, 520–525, doi:10.1590/1519-6984.23014.
30. Lacerda, L.P.; Malaquias, G.; Peron, A.P. Antiproliferative action of aqueous extracts of *Hymenaea stigonocarpa* Mart. (Fabaceae) on the cell cycle of *Allium cepa* L. *Anais da Academia Brasileira de Ciencias* **2014**, *86*, 1147–1150, doi:10.1590/0001-3765201420130163.
31. Bosquiroli, L.S.S.; dos Ferreira, A.C.S.; Farias, K.S.; da Costa, E.C.; de Matos, M.F.C.; Kadri, M.C.T.; Rizk, Y.S.; Alves, F.M.; Perdomo, R.T.; Carollo, C.A.; et al. In vitro antileishmania activity of sesquiterpene-rich essential oils from *Nectandra* species. *Pharmaceutical Biology* **2017**, *55*, 2285–2291, doi:10.1080/13880209.2017.1407803.
32. Costa, É.R.; Louro, G.M.; Simionatto, S.; Vasconcelos, N.G.; Cardoso, C.A.L.; Mallmann, V.; da Silva, R.C.L.; Matos, M. de F.C.; Pizzuti, L.; Santiago, E.F.; et al. Chemical Composition, Antitumoral and Antibacterial Activities of Essential Oils from Leaves and Stem Bark of *Nectandra lanceolata* (Lauraceae). *Journal of Essential Oil-Bearing Plants* **2017**, *20*, 1184–1195, doi:10.1080/0972060X.2017.1394223.
33. Farias, K.S.; Kato, N.N.; Boaretto, A.G.; Weber, J.I.; Brust, F.R.; Alves, F.M.; Tasca, T.; Macedo, A.J.; Silva, D.B.; Carollo, C.A. *Nectandra* as a renewable source for (+)-α-bisabolol, an antibiofilm and anti-*Trichomonas vaginalis* compound. *Fitoterapia* **2019**, *136*, doi:10.1016/j.fitote.2019.104179.
34. Santos, F. V.; Colus, I.M.S.; Silva, M.A.; Vilegas, W.; Varanda, E.A. Assessment of DNA damage by extracts and fractions of *Strychnos pseudoquina*, a Brazilian medicinal plant with antiulcerogenic activity. *Food and Chemical Toxicology* **2006**, *44*, 1585–1589, doi:10.1016/j.fct.2006.03.012.
35. Reichert, C.L.; Silva, D.B.; Carollo, C.A.; Weffort-Santos, A.M.; Santos, C.A.M. Metabolic profiling and correlation analysis for the determination of killer compounds of proliferating and clonogenic HRT-18 colon cancer cells from *Lafoensia pacari*. *Journal of Ethnopharmacology* **2018**, *224*, 541–552, doi:10.1016/j.jep.2018.06.021.
36. Da Silva Marcondes, D.B.; Reichert, C.L.; De Andrade, L.F.; De Moraes Santos, C.A.; Weffort-Santos, A.M. Cytotoxicity and apoptogenic effects of *Lafoensia pacari*. *Journal of Ethnopharmacology* **2014**, *157*, 243–250, doi:10.1016/j.jep.2014.09.018.
37. Gasca, C.A.; Castillo, W.O.; Takahashi, C.S.; Fagg, C.W.; Magalhães, P.O.; Fonseca-Bazzo, Y.M.; Silveira, D. Assessment of

anti-cholinesterase activity and cytotoxicity of cagaita (*Eugenia dysenterica*) leaves. *Food and Chemical Toxicology* **2017**, *109*, 996–1002, doi:10.1016/j.fct.2017.02.032.
38. Vieira, P.M.; Veronezi, E.; Silva, C.R.; Chen-Chen, L. Detection of genotoxic, cytotoxic, and protective activities of *Eugenia dysenterica* DC. (Myrtaceae) in mice. *Journal of Medicinal Food* **2012**, *15*, 563–567, doi:10.1089/jmf.2011.0270.
39. da Silva, V.P.; Alves, C.C.F.; Miranda, M.L.D.; Bretanha, L.C.; Balleste, M.P.; Micke, G.A.; Silveira, E.V.; Martins, C.H.G.; Ambrosio, M.A.L.V.; de Souza Silva, T.; et al. Chemical composition and in vitro leishmanicidal, antibacterial and cytotoxic activities of essential oils of the Myrtaceae family occurring in the Cerrado biome. *Industrial Crops and Products* **2018**, *123*, 638–645, doi:10.1016/j.indcrop.2018.07.033.
40. Serpeloni, J.M.; Leal Specian, A.F.; Ribeiro, D.L.; Tuttis, K.; Vilegas, W.; Martínez-López, W.; Dokkedal, A.L.; Saldanha, L.L.; De Syllos Cólus, I.M.; Varanda, E.A. Antimutagenicity and induction of antioxidant defense by flavonoid rich extract of *Myrcia bella* Cambess. in normal and tumor gastric cells. *Journal of Ethnopharmacology* **2015**, *176*, 345–355, doi:10.1016/j.jep.2015.11.003.
41. Machado, A.J.T.; Santos, A.T.L.; Martins, G.M.A.B.; Cruz, R.P.; Costa, M. do S.; Campina, F.F.; Freitas, M.A.; Bezerra, C.F.; Leal, A.L.A.B.; Carneiro, J.N.P.; et al. Antiparasitic effect of the *Psidium guajava* L. (guava) and *Psidium brownianum* MART. EX DC. (araçá-de-veado) extracts. *Food and Chemical Toxicology* **2018**, *119*, 275–280, doi:10.1016/j.fct.2018.03.018.
42. Napolitano, D.R.; Mineo, J.R.; De Souza, M.A.; De Paula, J.E.; Espindola, L.S.; Espindola, F.S. Down-modulation of nitric oxide production in murine macrophages treated with crude plant extracts from the Brazilian Cerrado. *Journal of Ethnopharmacology* **2005**, *99*, 37–41, doi:10.1016/j.jep.2005.01.059.
43. Elias, S.; Salles, P.; De Paula, J.; Simeoni, L.; Silveira, D.; Guerra, E.; Motoyama, A. Cytotoxic effect of *Pouteria torta* leaf extracts on human oral and breast carcinomas cell lines. *Journal of Cancer Research and Therapeutics* **2013**, *9*, 601–606, doi:10.4103/0973-1482.126454.
44. Funari, C.S.; Passalacqua, T.G.; Rinaldo, D.; Napolitano, A.; Festa, M.; Capasso, A.; Piacente, S.; Pizza, C.; Young, M.C.M.; Durigan, G.; et al. Interconverting flavanone glucosides and other phenolic compounds in *Lippia salviaefolia* Cham. ethanol extracts. *Phytochemistry* **2011**, *72*, 2052–2061, doi:10.1016/j.phytochem.2011.07.004.
45. Munari, C.C.; Resende, F.A.; Alves, J.M.; De Sousa, J.P.B.; Bastos, J.K.; Tavares, D.C. Mutagenicity and antimutagenicity of *Baccharis dracunculifolia* extract in chromosomal aberration assays in Chinese hamster ovary cells. *Planta Medica* **2008**, *74*, 1363–1367, doi:10.1055/s-2008-1081306.
46. Resende, F.A.; Nogueira, C.H.; Espanha, L.G.; Boldrin, P.K.; Oliveira-Höhne, A.P.; Santoro de Camargo, M.; Quintino da Rocha, C.; Vilegas, W.; Varanda, E.A. In vitro toxicological assessment of *Arrabidaea brachypoda* (DC.) Bureau: Mutagenicity and estrogenicity studies. *Regulatory Toxicology and Pharmacology* **2017**, *90*, 29–35, doi:10.1016/j.yrtph.2017.08.010.
47. Beserra, A.M.S. e. S.; Vilegas, W.; Tangerina, M.M.P.; Ascêncio, S.D.; Soares, I.M.; Pavan, E.; Damazo, A.S.; Ribeiro, R.V.; Martins, D.T. de O. Chemical characterisation and toxicity assessment in vitro and in vivo of the hydroethanolic extract of *Terminalia argentea* Mart. leaves. *Journal of Ethnopharmacology* **2018**, *227*, 56–68, doi:10.1016/j.jep.2018.08.025.
48. Santos, F. V.; Nasser, A.L.M.; Biso, F.I.; Moreira, L.M.; Santos, V.J.S.V.; Vilegas, W.; Varanda, E.A. Genotoxicity of polar and apolar extracts obtained from *Qualea multiflora* and *Qualea grandiflora*. *Journal of Ethnopharmacology* **2011**, *138*, 105–110, doi:10.1016/j.jep.2011.08.062.
49. Biso, F.I.; Rodrigues, C.M.; Rinaldo, D.; Reis, M.B. dos; Bernardi, C.C.; de Mattos, J.C.P.; Caldeira-de-Araújo, A.; Vilegas, W.; Cólus, I.M. de S.; Varanda, E.A. Assessment of DNA damage induced by extracts, fractions and isolated compounds of *Davilla nitida* and *Davilla elliptica* (Dilleniaceae). *Mutation Research - Genetic Toxicology and Environmental Mutagenesis* **2010**, *702*, 92–99, doi:10.1016/j.mrgentox.2010.07.011.
50. Cardoso, C.R.P.; Cólus, I.M. de S.; Bernardi, C.C.; Sannomiya, M.; Vilegas, W.; Varanda, E.A. Mutagenic activity promoted by amentoflavone and methanolic extract of *Byrsonima crassa* Niedenzu. *Toxicology* **2006**, *225*, 55–63, doi:10.1016/j.tox.2006.05.003.
51. Santos, F. V.; Tubaldini, F.R.; Cólus, I.M.S.; Andréo, M.A.; Bauab, T.M.; Leite, C.Q.F.; Vilegas, W.; Varanda, E.A. Mutagenicity of *Mouriri pusa* Gardner and *Mouriri elliptica* Martius. *Food and Chemical Toxicology* **2008**, *46*, 2721–2727, doi:10.1016/j.fct.2008.04.028.
52. Avelar Amado, P.; Fonsêca Castro, A.H.; Samúdio Santos Zanuncio, V.; Stein, V.C.; Brentan da Silva, D.; Alves Rodrigues dos Santos Lima, L. Assessment of allelopathic, cytotoxic, genotoxic and antigenotoxic potential of *Smilax brasiliensis* Sprengel leaves. *Ecotoxicology and Environmental Safety* **2020**, *192*, doi:10.1016/j.ecoenv.2020.110310.
53. Ferreira, S.A.; Guimarães, A.G.; Ferrari, F.C.; Carneiro, C.M.; de Paiva, N.C.N.; Guimarães, D.A.S. Assessment of acute toxicity of the ethanolic extract of *Lychnophora pinaster* (Brazilian arnica). *Revista Brasileira de Farmacognosia* **2014**, *24*, 553–560, doi:10.1016/j.bjp.2014.09.005.
54. Ferrari, F.C.; Grabe-Guimarães, A.; Carneiro, C.M.; de Souza, M.R.; Ferreira, L.C.; de Oliveira, T.T.; Saúde-Guimarães, D.A. Toxicological evaluation of ethanolic extract of *Lychnophora trichocarpha*, Brazilian arnica. *Revista Brasileira de Farmacognosia* **2012**, *22*, 1104–1110, doi:10.1590/S0102-695X2012005000089.
55. Féres, C.A.O.; Madalosso, R.C.; Rocha, O.A.; Leite, J.P.V.; Guimarães, T.M.D.P.; Toledo, V.P.P.; Tagliati, C.A. Acute and chronic toxicological studies of *Dimorphandra mollis* in experimental animals. *Journal of Ethnopharmacology* **2006**, *108*, 450–456,

doi:10.1016/j.jep.2006.06.002.
56. Da Cunha, L.C.; De Paula, J.R.; De Sá, V.A.; E Amorim, M.E.D.P.; Barros, I.C.M.; Brito, L.A.B.; Da Silveira, N. Acute toxicity of *Brosimum gaudichaudii* Trécul. root extract in mice: Determination of both approximate and median lethal doses. *Brazilian Journal of Pharmacognosy* **2008**, *18*, 532–538, doi:10.1590/S0102-695X2008000400006.
57. Araújo, M.C. de P.M.; Barcellos, N.M.S.; Vieira, P.M. de A.; Gouveia, T.M.; Guerra, M.O.; Peters, V.M.; Saúde-Guimarães, D.A. Acute and sub chronic toxicity study of aqueous extract from the leaves and branches of *Campomanesia velutina* (Cambess) O. Berg. *Journal of Ethnopharmacology* **2017**, *201*, 17–25, doi:10.1016/j.jep.2017.02.043.
58. da Silva Moreira, S.; Tamashiro, L.K.; Jorge, B.C.; da Silva Balin, P.; Heredia-Vieira, S.C.; de Almeida, G.L.; Cardoso, C.A.L.; Kassuya, C.A.L.; Arena, A.C. Toxicological safety evaluation in acute and 28-day studies of aqueous extract from *Serjania marginata* Casar. (Sapindaceae) leaves in rats. *Journal of Ethnopharmacology* **2019**, *231*, 197–204, doi:10.1016/j.jep.2018.11.024.
59. Pires, J.G.; Zabini, S.S.; Braga, A.S.; de Cássia Fabris, R.; de Andrade, F.B.; de Oliveira, R.C.; Magalhães, A.C. Hydroalcoholic extracts of *Myracrodruon urundeuva* All. and *Qualea grandiflora* Mart. leaves on *Streptococcus mutans* biofilm and tooth demineralization. *Archives of Oral Biology* **2018**, *91*, 17–22, doi:10.1016/j.archoralbio.2018.04.005.
60. Ribeiro, I.C. de O.; Mariano, E.G.A.; Careli, R.T.; Morais-Costa, F.; De Sant'Anna, F.M.; Pinto, M.S.; De Souza, M.R.; Duarte, E.R. Plants of the Cerrado with antimicrobial effects against *Staphylococcus* spp. and *Escherichia coli* from cattle. *BMC Veterinary Research* **2018**, *14*, doi:10.1186/s12917-018-1351-1.
61. Terezan, A.P.; Junqueira, J.G.M.; Wakui, V.G.; Kato, L.; Oliveira, C.M.A.; Martins, C.H.G.; Santiago, M.B.; Severino, V.G.P. Qualitative analysis of the acetogenins from *Annona coriacea* (Annonaceae) leaves by HPLC-Q-Orbitrap and their antibacterial potential against oral pathogens. *Natural product research* **2020**, 1–7, doi:10.1080/14786419.2020.1803312.
62. Xavier, M.N.; Alves, J.M.; Carneiro, N.S.; Souchie, E.L.; Da Silva, E.A.J.; Martins, C.H.G.; Ambrosio, M.A.L.V.; Egea, M.B.; Alves, C.C.F.; Miranda, M.L.D. Chemical composition from essential oil of *Cardiopetalum calophyllum* schltdl. (Annonaceae) and their antioxidant, antibacterial and antifungal activities. *Revista Virtual de Quimica* **2016**, *8*, 1433–1448, doi:10.21577/1984-6835.20160101.
63. Moraes, T. de M.; Rodrigues, C.M.; Kushima, H.; Bauab, T.M.; Villegas, W.; Pellizzon, C.H.; Brito, A.R.M.S.; Hiruma-Lima, C.A. *Hancornia speciosa*: Indications of gastroprotective, healing and anti-*Helicobacter pylori* actions. *Journal of Ethnopharmacology* **2008**, *120*, 161–168, doi:10.1016/j.jep.2008.08.001.
64. Ribeiro, D.A.; Damasceno, S.S.; Boligon, A.A.; Menezes, I.R.A. de; Souza, M.M. de A.; Costa, J.G.M. da Chemical profile and antimicrobial activity of *Secondatia floribunda* A. DC (Apocynaceae). *Asian Pacific Journal of Tropical Biomedicine* **2017**, *7*, 739–749, doi:10.1016/j.apjtb.2017.07.009.
65. Oliveira, D.M.; Furtado, F.B.; Gomes, A.A.S.; Belut, B.R.; Nascimento, E.A.; Morais, S.A.L.; Martins, C.H.G.; Santos, V.C.O.; Da Silva, C. V.; Teixeira, T.L.; et al. Chemical Constituents and Antileishmanial and Antibacterial Activities of Essential Oils from *Scheelea phalerata*. *ACS Omega* **2020**, *5*, 1363–1370, doi:10.1021/acsomega.9b01962.
66. Nobre, C.B.; de Sousa, E.O.; de Lima Silva, J.M.F.; Melo Coutinho, H.D.; da Costa, J.G.M. Chemical composition and antibacterial activity of fixed oils of *Mauritia flexuosa* and *Orbignya speciosa* associated with aminoglycosides. *European Journal of Integrative Medicine* **2018**, *23*, 84–89, doi:10.1016/j.eujim.2018.09.009.
67. Siqueira, E.P. De; Andrade, A.A.; Souza-, E.M. De; Ramos, J.P.; Kohlhoff, M.; Roberta, Y.; Nunes, F.; Veloso, M.; Campos, F.F.; Johann, S.; et al. In vitro antibacterial action on methicillin-susceptible (MSSA) and methicillin-resistant (MRSA) *Staphylococcus aureus* and antitumor potential of *Mauritia flexuosa* L. f. *Journal of Medicinal Plants Research* **2014**, *8*, 1408–1417, doi:10.5897/JMPR2014.5688.
68. Machado, T.D.B.; Leal, I.C.R.; Kuster, R.M.; Amaral, A.C.F.; Kokis, V.; De Silva, M.G.; Dos Santos, K.R.N. Brazilian phytopharmaceuticals - Evaluation against hospital bacteria. *Phytotherapy Research* **2005**, *19*, 519–525, doi:10.1002/ptr.1696.
69. Alviano, W.S.; Alviano, D.S.; Diniz, C.G.; Antoniolli, A.R.; Alviano, C.S.; Farias, L.M.; Carvalho, M.A.R.; Souza, M.M.G.; Bolognese, A.M. In vitro antioxidant potential of medicinal plant extracts and their activities against oral bacteria based on Brazilian folk medicine. *Archives of Oral Biology* **2008**, *53*, 545–552, doi:10.1016/j.archoralbio.2007.12.001.
70. de Jesus, G.S.; Micheletti, A.C.; Padilha, R.G.; de Souza de Paula, J.; Alves, F.M.; Leal, C.R.B.; Garcez, F.R.; Garcez, W.S.; Yoshida, N.C. Antimicrobial Potential of Essential Oils from Cerrado Plants against Multidrug-Resistant Foodborne Microorganisms. *Molecules (Basel, Switzerland)* **2020**, *25*, doi:10.3390/molecules25143296.
71. Carvalho, R.S.; Carollo, C.A.; de Magalhães, J.C.; Palumbo, J.M.C.; Boaretto, A.G.; Nunes e Sá, I.C.; Ferraz, A.C.; Lima, W.G.; de Siqueira, J.M.; Ferreira, J.M.S. Antibacterial and antifungal activities of phenolic compound-enriched ethyl acetate fraction from *Cochlospermum regium* (mart. Et. Schr.) Pilger roots: Mechanisms of action and synergism with tannin and gallic acid. *South African Journal of Botany* **2018**, *114*, 181–187, doi:10.1016/j.sajb.2017.11.010.
72. Arunachalam, K.; Damazo, A.S.; Pavan, E.; Oliveira, D.M.; Figueiredo, F. de F.; Machado, M.T.M.; Balogun, S.O.; Soares, I.M.; Barbosa, R. dos S.; Alvim, T. da C.; et al. *Cochlospermum regium* (Mart. ex Schrank) Pilg.: Evaluation of chemical profile, gastroprotective activity and mechanism of action of hydroethanolic extract of its xylopodium in acute and chronic experimental models. *Journal of Ethnopharmacology* **2019**, *233*, 101–114, doi:10.1016/j.jep.2019.01.002.
73. Galvão, F. de O.; Dantas, F.G. da S.; Santos, C.R. de L.; Marchioro, S.B.; Cardoso, C.A.L.; Wender, H.; Sangalli, A.; Almeida-Apolonio, A.A. de; Oliveira, K.M.P. de *Cochlospermum regium* (Schrank) pilger leaf extract inhibit methicillin-resistant

Staphylococcus aureus biofilm formation. *Journal of Ethnopharmacology* **2020**, *261*, doi:10.1016/j.jep.2020.113167.
74. Leme, D.E.M.; Rodrigues, A.B.; Almeida-Apolonio, A.A. De; Dantas, F.G.D.S.; Negri, M.F.N.; Svidzinski, T.I.E.; Mota, J.D.S.; Cardoso, C.A.L.; Oliveira, K.M.P. De In Vitro Control of Uropathogenic Microorganisms with the Ethanolic Extract from the Leaves of *Cochlospermum regium* (Schrank) Pilger. *Evidence-based Complementary and Alternative Medicine* **2017**, *2017*, doi:10.1155/2017/4687154.
75. De Toledo, C.E.M.; Britta, E.A.; Ceole, L.F.; Silva, E.R.; De Mello, J.C.P.; Dias Filho, B.P.; Nakamura, C.V.; Ueda-Nakamura, T. Antimicrobial and cytotoxic activities of medicinal plants of the Brazilian cerrado, using Brazilian cachaa as extractor liquid. *Journal of Ethnopharmacology* **2011**, *133*, 420–425, doi:10.1016/j.jep.2010.10.021.
76. Ribeiro, I.C. de O.; Mariano, E.G.A.; Careli, R.T.; Morais-Costa, F.; De Sant'Anna, F.M.; Pinto, M.S.; De Souza, M.R.; Duarte, E.R. Plants of the Cerrado with antimicrobial effects against *Staphylococcus* spp. and *Escherichia coli* from cattle. *BMC Veterinary Research* **2018**, *14*, 1–10, doi:10.1186/s12917-018-1351-1.
77. Amaral, L.F.B.; Moriel, P.; Foglio, M.A.; Mazzola, P.G. *Caryocar brasiliense* supercritical CO2 extract possesses antimicrobial and antioxidant properties useful for personal care products. *BMC Complementary and Alternative Medicine* **2014**, *14*, doi:10.1186/1472-6882-14-73.
78. Kushima, H.; Nishijima, C.M.; Rodrigues, C.M.; Rinaldo, D.; Sassá, M.F.; Bauab, T.M.; Stasi, L.C. Di; Carlos, I.Z.; Brito, A.R.M.S.; Vilegas, W.; et al. Davilla elliptica and Davilla nitida: Gastroprotective, anti-inflammatory immunomodulatory and anti-*Helicobacter pylori* action. *Journal of Ethnopharmacology* **2009**, *123*, 430–438, doi:10.1016/j.jep.2009.03.031.
79. Violante, I.M.P.; Hamerski, L.; Silva Garcez, W.; Batista, A.L.; Chang, M.R.; Pott, V.J.; Rodrigues Garcez, F. Antimicrobial Activity of Some Medicinal Plants From the Cerrado of the Central- Western Region of Brazil. *Brazilian Journal of Microbiology* **2012**, 1302–1308.
80. Lima, Z.P.; Calvo, T.R.; Silva, E.F.; Pellizzon, C.H.; Vilegas, W.; Brito, A.R.M.S.; Bauab, T.M.; Hiruma-Lima, C.A. Brazilian medicinal plant acts on prostaglandin level and *Helicobacter pylori*. *Journal of Medicinal Food* **2008**, *11*, 701–708, doi:10.1089/jmf.2007.0676.
81. Araújo, F.M.; Dantas, M.C.S.M.; e Silva, L.S.; Aona, L.Y.S.; Tavares, I.F.; de Souza-Neta, L.C. Antibacterial activity and chemical composition of the essential oil of *Croton heliotropiifolius* Kunth from Amargosa, Bahia, Brazil. *Industrial Crops and Products* **2017**, *105*, 203–206, doi:10.1016/j.indcrop.2017.05.016.
82. De Almeida Alves, T.M.; Fonseca Silva, A.; Brandão, M.; Mesquita Grandi, T.S.; Smânia, E.D.F.A.; Smânia, A.; Zani, C.L. Biological Screening of Brazilian Medicinal Plants. *Memorias do Instituto Oswaldo Cruz* **2000**, *95*, 367–373, doi:10.1590/s0074-02762000000300012.
83. de Oliveira Cartaxo-Furtado, N.A.; Brandão, D.O.; de Lima Ramos Júnior, F.J.; Silva, K.M.A.; Macêdo, R.O. Investigation of thermal and kinetic behavior of the *Stryphnodendron adstringens* dry extract with antimicrobial activity. *Journal of Thermal Analysis and Calorimetry* **2019**, *138*, 3781–3788, doi:10.1007/s10973-019-08047-5.
84. Sedano-Partida, M.D.; Santos, K.P. dos; Sala-Carvalho, W.R.; Silva-Luz, C.L.; Furlan, C.M. Anti-HIV-1 and antibacterial potential of H*yptis radicans* (Pohl) Harley & J.F.B. Pastore and *Hyptis multibracteata* Benth. (Lamiaceae). *Journal of Herbal Medicine* **2020**, *20*, 100328, doi:10.1016/j.hermed.2019.100328.
85. Zellner, B.D.; Amorim, A.C.L.; de Miranda, A.L.P.; Alves, R.J.V.; Barbosa, J.P.; da Costa, G.L.; Rezende, C.M. Screening of the odour-activity and bioactivity of the essential oils of leaves and flowers of *Hyptis passerina* Mart. from the Brazilian Cerrado. *Journal of the Brazilian Chemical Society* **2009**, *20*, 322–332, doi:10.1590/S0103-50532009000200018.
86. Bonamin, F.; Moraes, T.M.; Kushima, H.; Silva, M.A.; Rozza, A.L.; Pellizzon, C.H.; Bauab, T.M.; Rocha, L.R.M.H.; Vilegas, W.; Hiruma-Lima, C.A. Can a S*trychnos* species be used as antiulcer agent? Ulcer healing action from alkaloid fraction of *Strychnos pseudoquina* St. Hil. (Loganiaceae). *Journal of Ethnopharmacology* **2011**, *138*, 47–52, doi:10.1016/j.jep.2011.08.020.
87. Santos, R.C.; Kushima, H.; Rodrigues, C.M.; Sannomiya, M.; Rocha, L.R.M.; Bauab, T.M.; Tamashiro, J.; Vilegas, W.; Hiruma-Lima, C.A. *Byrsonima intermedia* A. Juss.: Gastric and duodenal anti-ulcer, antimicrobial and antidiarrheal effects in experimental rodent models. *Journal of Ethnopharmacology* **2012**, *140*, 203–212, doi:10.1016/j.jep.2011.12.008.
88. Moleiro, F.C.; Andreo, M.A.; Santos, R. de C. dos; Moraes, T. de M.; Rodrigues, C.M.; Carli, C.B. de A.; Lopes, F.C.M.; Pellizzon, C.H.; Carlos, I.Z.; Bauab, T.M.; et al. *Mouriri elliptica*: Validation of gastroprotective, healing and anti-*Helicobacter pylori* effects. *Journal of Ethnopharmacology* **2009**, *123*, 359–368, doi:10.1016/j.jep.2009.03.040.
89. Daniel Daza, L.; Fujita, A.; Granato, D.; Silvia Fávaro-Trindade, C.; Inés Genovese, M. Functional properties of encapsulated Cagaita (*Eugenia dysenterica* DC.) fruit extract. *Food Bioscience* **2017**, *18*, 15–21, doi:10.1016/j.fbio.2017.03.003.
90. Sousa, R.M.F.; de Morais, S.A.L.; Vieira, R.B.K.; Napolitano, D.R.; Guzman, V.B.; Moraes, T.S.; Cunha, L.C.S.; Martins, C.H.G.; Chang, R.; de Aquino, F.J.T.; et al. Chemical composition, cytotoxic, and antibacterial activity of the essential oil from E*ugenia calycina* Cambess. leaves against oral bacteria. *Industrial Crops and Products* **2015**, *65*, 71–78, doi:10.1016/j.indcrop.2014.11.050.
91. Carneiro, N.S.; Alves, C.C.F.; Alves, J.M.; Egea, M.B.; Martins, C.H.G.; Silva, T.S.; Bretanha, L.C.; Balleste, M.P.; Micke, G.A.; Silveira, E. V.; et al. Chemical composition, antioxidant and antibacterial activities of essential oils from leaves and flowers of *Eugenia klotzschiana* Berg (Myrtaceae). *Anais da Academia Brasileira de Ciencias* **2017**, *89*, 1907–1915, doi:10.1590/0001-3765201720160652.
92. da Silva, V.P.; Alves, C.C.F.; Miranda, M.L.D.; Bretanha, L.C.; Balleste, M.P.; Micke, G.A.; Silveira, E.V.; Martins, C.H.G.;

Ambrosio, M.A.L.V.; de Souza Silva, T.; et al. Chemical composition and in vitro leishmanicidal, antibacterial and cytotoxic activities of essential oils of the Myrtaceae family occurring in the Cerrado biome. *Industrial Crops and Products* **2018**, *123*, 638–645, doi:10.1016/j.indcrop.2018.07.033.

93. Santos, C. dos; Galaverna, R.S.; Angolini, C.F.F.; Nunes, V.V.A.; de Almeida, L.F.R.; Ruiz, A.L.T.G.; de Carvalho, J.E.; Duarte, R.M.T.; Duarte, M.C.T.; Eberlin, M.N. Antioxidative, antiproliferative and antimicrobial activities of phenolic compounds from three *Myrcia* species. *Molecules* **2018**, *23*, 1–12, doi:10.3390/molecules23050986.
94. Zandoná, G.P.; Bagatini, L.; Woloszyn, N.; de Souza Cardoso, J.; Hoffmann, J.F.; Moroni, L.S.; Stefanello, F.M.; Junges, A.; Rombaldi, C.V. Extraction and characterization of phytochemical compounds from araçazeiro (*Psidium cattleianum*) leaf: Putative antioxidant and antimicrobial properties. *Food Research International* **2020**, *137*, 109573, doi:10.1016/j.foodres.2020.109573.
95. do Nascimento, K.F.; Moreira, F.M.F.; Alencar Santos, J.; Kassuya, C.A.L.; Croda, J.H.R.; Cardoso, C.A.L.; Vieira, M. do C.; Góis Ruiz, A.L.T.; Ann Foglio, M.; de Carvalho, J.E.; et al. Antioxidant, anti-inflammatory, antiproliferative and antimycobacterial activities of the essential oil of *Psidium guineense* Sw. and spathulenol. *Journal of Ethnopharmacology* **2018**, *210*, 351–358, doi:10.1016/j.jep.2017.08.030.
96. Durães, E.R.B.; Clementino, C. de O.; Fari, L.R.; Ramos, L.M.; Oliveira, M.S.; Paula, J.A.M. de; Naves, P.L.F. Phytochemical study, toxicity and antimicrobial activity of *Psidium myrsinites* DC. (Myrtaceae) leaves. *Bioscience Journal* **2017**, 1305–1313, doi:10.14393/bj-v33n5a2017-34763.
97. Sá, S.; Chaul, L.T.; Alves, V.F.; Fiuza, T.S.; Tresvenzol, L.M.F.; Vaz, B.G.; Ferri, P.H.; Borges, L.L.; Paula, J.R. Phytochemistry and antimicrobial activity of *Campomanesia adamantium*. *Revista Brasileira de Farmacognosia* **2018**, *28*, 303–311, doi:10.1016/j.bjp.2018.02.008.
98. Pavan, F.R.; Leite, C.Q.F.; Coelho, R.G.; Coutinho, I.D.; Honda, N.K.; Cardoso, C.A.L.; Vilegas, W.; De Andrade Leite, S.R.; Sato, D.N. Evaluation of anti-*Mycobacterium tuberculosis* activity of *Campomanesia adamantium* (Myrtaceae). *Quimica Nova* **2009**, *32*, 1222–1226, doi:10.1590/S0100-40422009000500026.
99. Arunachalam, K.; Ascêncio, S.D.; Soares, I.M.; Souza Aguiar, R.W.; Da Silva, L.I.; De Oliveira, R.G.; Balogun, S.O.; De Oliveira Martins, D.T. *Gallesia integrifolia* (Spreng.) Harms: In vitro and in vivo antibacterial activities and mode of action. *Journal of Ethnopharmacology* **2016**, *184*, 128–137, doi:10.1016/j.jep.2016.03.005.
100. Périco, L.L.; Heredia-Vieira, S.C.; Beserra, F.P.; De Cássia Dos Santos, R.; Weiss, M.B.; Resende, F.A.; Dos Santos Ramos, M.A.; Bonifácio, B.V.; Bauab, T.M.; Varanda, E.A.; et al. Does the gastroprotective action of a medicinal plant ensure healing effects? An integrative study of the biological effects of *Serjania marginata* Casar. (Sapindaceae) in rats. *Journal of Ethnopharmacology* **2015**, *172*, 312–324, doi:10.1016/j.jep.2015.06.025.
101. de Melo, D.C.; Miranda, M.L.D.; Ferreira Júnior, W.G.; de Andrade, P.M.; Alcoba, A.E.T.; Silva, T. de S.; Cazal, C. de M.; Martins, C.H.G. Anticariogenic and antimycobacterial activities of the essential oil of *Siparuna guianensis* Aublet (Siparunaceae). *Orbital* **2017**, *9*, 55–60, doi:10.17807/orbital.v0i0.930.
102. Mazzolin, L.P.; Nasser, A.L.M.; Moraes, T.M.; Santos, R.C.; Nishijima, C.M.; Santos, F.V.; Varanda, E.A.; Bauab, T.M.; da Rocha, L.R.M.; Di Stasi, L.C.; et al. *Qualea parviflora* Mart.: An integrative study to validate the gastroprotective, antidiarrheal, antihemorragic and mutagenic action. *Journal of Ethnopharmacology* **2010**, *127*, 508–514, doi:10.1016/j.jep.2009.10.005.
103. de Almeida-Apolonio, A.A.; Cupozak-Pinheiro, W.J.; da Silva Dantas, F.G.; Mattos, K.; Cardoso, C.A.L.; Negri, M.; Chang, M.R.; de Oliveira, K.M.P. *Myracrodruon urundeuva* All. aqueous extract: A promising mouthwash for the prevention of oral candidiasis in HIV/AIDS patients. *Industrial Crops and Products* **2020**, *145*, 111950, doi:10.1016/j.indcrop.2019.111950.
104. Duarte, M.C.T.; Figueira, G.M.; Sartoratto, A.; Rehder, V.L.G.; Delarmelina, C. Anti-*Candida* activity of Brazilian medicinal plants. *Journal of Ethnopharmacology* **2005**, *97*, 305–311, doi:10.1016/j.jep.2004.11.016.
105. Pereira, A.M.S.; Hernandes, C.; Pereira, S.I.V.; Bertoni, B.W.; França, S.C.; Pereira, P.S.; Taleb-Contini, S.H. Evaluation of anticandidal and antioxidant activities of phenolic compounds from *Pyrostegia venusta* (Ker Gawl.) Miers. *Chemico-Biological Interactions* **2014**, *224*, 136–141, doi:10.1016/j.cbi.2014.10.023.
106. Leme, D.E.M.; Rodrigues, A.B.; de Almeida-Apolonio, A.A.; Dantas, F.G. da S.; Negri, M.F.N.; Svidzinski, T.I.E.; Mota, J. da S.; Cardoso, C.A.L.; de Oliveira, K.M.P. In Vitro Control of Uropathogenic Microorganisms with the Ethanolic Extract from the Leaves of *Cochlospermum regium* (Schrank) Pilger. *Evidence-based complementary and alternative medicine : eCAM* **2017**, *2017*, 4687154, doi:10.1155/2017/4687154.
107. Albernaz, L.C.; de Paula, J.E.; Romero, G.A.S.; Silva, M. do R.R.; Grellier, P.; Mambu, L.; Espindola, L.S. Investigation of plant extracts in traditional medicine of the Brazilian Cerrado against protozoans and yeasts. *Journal of Ethnopharmacology* **2010**, *131*, 116–121, doi:10.1016/j.jep.2010.06.011.
108. Rodrigues de Araújo, A.; Iles, B.; de Melo Nogueira, K.; Dias, J. do N.; Plácido, A.; Rodrigues, A.; Albuquerque, P.; Silva-Pereira, I.; Socodatto, R.; Portugal, C.C.; et al. Antifungal and anti-inflammatory potential of eschweilenol C-rich fraction derived from *Terminalia fagifolia* Mart. *Journal of Ethnopharmacology* **2019**, *240*, 111941, doi:10.1016/j.jep.2019.111941.
109. Da Costa, R.C.; Santana, D.B.; Araújo, R.M.; De Paula, J.E.; Do Nascimento, P.C.; Lopes, N.P.; Braz-Filho, R.; Espindola, L.S. Discovery of the rapanone and suberonone mixture as a motif for leishmanicidal and antifungal applications. *Bioorganic and Medicinal Chemistry* **2014**, *22*, 135–140, doi:10.1016/j.bmc.2013.11.044.

110. Mendes De Toledo, C.E.; Santos, P.R.; Palazzo De Mello, J.C.; Dias Filho, B.P.; Nakamura, C.V.; Ueda-Nakamura, T. Antifungal Properties of Crude Extracts, Fractions, and Purified Compounds from Bark of *Curatella americana* L. (Dilleniaceae) against *Candida* Species. *Evidence-based Complementary and Alternative Medicine* **2015**, *2015*, doi:10.1155/2015/673962.
111. Santana, D.B.; da Costa, R.C.; Araújo, R.M.; de Paula, J.E.; Silveira, E.R.; Braz-Filho, R.; Espindola, L.S. Activity of Fabaceae species extracts against fungi and *Leishmania*: Vatacarpan as a novel potent anti-*Candida* agent. *Revista Brasileira de Farmacognosia* **2015**, *25*, 401–406, doi:10.1016/j.bjp.2015.07.012.
112. De Souza, A.C.M.; Kato, L.; Da Silva, C.C.; Cidade, A.F.; De Oliveira, C.M.A.; Silva, M. do R.R. Antimicrobial activity of *Hymenaea martiana* towards dermatophytes and *Cryptococcus neoformans*. *Mycoses* **2010**, *53*, 500–503, doi:10.1111/j.1439-0507.2009.01740.x.
113. do Carmo Silva, L.; Miranda, M.A.C.M.; de Freitas, J.V.; Ferreira, S.F.A.; de Oliveira Lima, E.C.; de Oliveira, C.M.A.; Kato, L.; Terezan, A.P.; Rodriguez, A.F.R.; Faria, F.S.E.D.V.; et al. Antifungal activity of Copaíba resin oil in solution and nanoemulsion against *Paracoccidioides* spp. *Brazilian Journal of Microbiology* **2020**, *51*, 125–134, doi:10.1007/s42770-019-00201-3.
114. Martins, C.D.M.; De Morais, S.A.L.; Martins, M.M.; Cunha, L.C.S.; Da Silva, C. V.; Martins, C.H.G.; Leandro, L.F.; De Oliveira, A.; De Aquino, F.J.T.; Do Nascimento, E.A.; et al. Chemical Composition, Antifungal, and Cytotoxicity Activities of *Inga laurina* (Sw.) Willd Leaves. *Scientific World Journal* **2019**, *2019*, doi:10.1155/2019/9423658.
115. Silva, F.M. e; De Paula, J.E.; Espindola, L.S. Evaluation of the antifungal potential of Brazilian Cerrado medicinal plants. *Mycoses* **2009**, *52*, 511–517, doi:10.1111/j.1439-0507.2008.01647.x.
116. Oliveira, D.M.; Silva, T.F.R.; Martins, M.M.; de Morais, S.A.L.; Chang, R.; de Aquino, F.J.T.; da Silva, C. V.; Teixeira, T.L.; Martins, C.H.G.; Moraes, T.S.; et al. Antifungal and cytotoxicity activities of *Banisteriopsis argyrophylla* leaves. *Journal of Pharmacy and Pharmacology* **2018**, *70*, 1541–1552, doi:10.1111/jphp.12996.
117. Nunes, B.C.; Martins, M.M.; Chang, R.; Morais, S.A.L.; Nascimento, E.A.; de Oliveira, A.; Cunha, L.C.S.; da Silva, C. V.; Teixeira, T.L.; Ambrósio, M.A.L.V.; et al. Antimicrobial activity, cytotoxicity and selectivity index of *Banisteriopsis laevifolia* (A. Juss.) B. Gates leaves. *Industrial Crops and Products* **2016**, *92*, 277–289, doi:10.1016/j.indcrop.2016.08.016.
118. Correia, A.F.; Silveira, D.; Fonseca-Bazzo, Y.M.; Magalhães, P.O.; Fagg, C.W.; da Silva, E.C.; Gomes, S.M.; Gandolfi, L.; Pratesi, R.; de Medeiros Nóbrega, Y.K. Activity of crude extracts from Brazilian cerrado plants against clinically relevant *Candida* species. *BMC Complementary and Alternative Medicine* **2016**, *16*, doi:10.1186/s12906-016-1164-3.
119. Costa, T.R.; Fernandes, O.F.L.; Santos, S.C.; Oliveira, C.M.A.; Lião, L.M.; Ferri, P.H.; Paula, J.R.; Ferreira, H.D.; Sales, B.H.N.; Silva, M.D.R.R. Antifungal activity of volatile constituents of *Eugenia dysenterica* leaf oil. *Journal of Ethnopharmacology* **2000**, *72*, 111–117, doi:10.1016/S0378-8741(00)00214-2.
120. Ferreira, F.P.S.; Morais, S.R.; Bara, M.T.F.; Conceição, E.C.; Paula, J.R.; Thays C. Carvalhoe, B.G.V.; Costaf, H.B.; Romãof, W.; Rezende, M.H. *Eugenia calycina* Cambess extracts and their fractions: Their antimicrobial activity and the identification of major polar compounds using electrospray ionization FT-ICR mass spectrometry. *Journal of Pharmaceutical and Biomedical Analysis* **2014**, *99*, 89–96, doi:10.1016/j.jpba.2014.07.003.
121. Da Costa, R.C.; Santana, D.B.; Araújo, R.M.; De Paula, J.E.; Do Nascimento, P.C.; Lopes, N.P.; Braz-Filho, R.; Espindola, L.S. Discovery of the rapanone and suberonone mixture as a motif for leishmanicidal and antifungal applications. *Bioorganic and Medicinal Chemistry* **2014**, *22*, 135–140, doi:10.1016/j.bmc.2013.11.044.
122. Morais-Braga, M.F.B.; Sales, D.L.; Carneiro, J.N.P.; Machado, A.J.T.; dos Santos, A.T.L.; de Freitas, M.A.; Martins, G.M. de A.B.; Leite, N.F.; de Matos, Y.M.L.S.; Tintino, S.R.; et al. *Psidium guajava* L. and *Psidium brownianum* Mart ex DC.: Chemical composition and anti - *Candida* effect in association with fluconazole. *Microbial Pathogenesis* **2016**, *95*, 200–207, doi:10.1016/j.micpath.2016.04.013.
123. De Assis, P.A.; Theodoro, P.N.E.T.; De Paula, J.E.; Araújo, A.J.; Costa-Lotufo, L. V.; Michel, S.; Grougnet, R.; Kritsanida, M.; Espindola, L.S. Antifungal ether diglycosides from *Matayba guianensis* Aublet. *Bioorganic and Medicinal Chemistry Letters* **2014**, *24*, 1414–1416, doi:10.1016/j.bmcl.2014.01.022.
124. Cecílio, A.B.; Faria, D.B. De; Oliveira, P.D.C.; Caldas, S.; Oliveira, D.A. De; Sobral, M.E.G.; Duarte, M.G.R.; Moreira, C.P.D.S.; Silva, C.G.; Almeida, V.L. De Screening of Brazilian medicinal plants for antiviral activity against rotavirus. *Journal of Ethnopharmacology* **2012**, *141*, 975–981, doi:10.1016/j.jep.2012.03.031.
125. Brandão, G.C.; Kroon, E.G.; Santos, J.R. dos; Stehmann, J.R.; Lombardi, J.A.; Oliveira, A.B. de Antiviral activity of plants occurring in the state of minas gerais (Brazil): Part III. *Journal of Chemical and Pharmaceutical Research* **2011**, *3*, 223–236.
126. Kohn, K.L.; Foglio, M.A.; Rodrigues, R.A.F.; Sousa, I.M.O.; Martini, M.C.; Padilha, M.A.; Lima, N.D. de.; Arns, C.W. In-vitro Antiviral of The Brazilian Cerrado against the Avian. *Brazilian Journal of Poultry Science.* **2015**, *17*, 275–280.
127. Brandão, G.C.; Kroon, E.G.; dos Santos, J.R.; Stehmann, J.R.; Lombardi, J.A.; de Oliveira, A.B. Antiviral activities of plants occurring in the state of Minas Gerais, Brazil. Part 2. Screening Bignoniaceae species. *Revista Brasileira de Farmacognosia* **2010**, *20*, 742–750, doi:10.1590/S0102-695X2010005000035.
128. Brandão, G.C.; Kroon, E.G.; Santos, J.R. dos; Stehmann, J.R.; Lombardi, J.A.; Oliveira, A.B. de Antiviral activity of Bignoniaceae species occurring in the state of Minas Gerais (Brazil): Part 1. *Letters in Applied Microbiology* **2010**, *51*, 469–476, doi:10.1111/j.1472-765X.2010.02924.x.

129. Cecílio, A.B.; Faria, D.B. De; Oliveira, P.D.C.; Caldas, S.; Oliveira, D.A. De; Sobral, M.E.G.; Duarte, M.G.R.; Moreira, C.P.D.S.; Silva, C.G.; Almeida, V.L. De Screening of Brazilian medicinal plants for antiviral activity against rotavirus. *Journal of Ethnopharmacology* **2012**, *141*, 975–981, doi:10.1016/j.jep.2012.03.031.
130. dos Santos, M.; Teixeira, T.R.; Santos, F.R. da S.; Lima, W.G.; Ferraz, A.C.; Silva, N.L.; Leite, F.J.; Siqueira, J.M.; Luyten, W.; de Castro, A.H.F.; et al. *Bauhinia holophylla* (Bong.) Steud. leaves-derived extracts as potent anti-dengue serotype 2. *Natural Product Research* **2019**, doi:10.1080/14786419.2019.1669030.
131. De Mesquita, M.L.; Desrivot, J.; Bories, C.; Fournet, A.; De Paula, J.E.; Grellier, P.; Espindola, L.S. Antileishmanial and trypanocidal activity of Brazilian Cerrado plants. *Memorias do Instituto Oswaldo Cruz* **2005**, *100*, 783–787, doi:10.1590/s0074-02762005000700019.
132. de Mesquita, M.L.; Grellier, P.; Mambu, L.; de Paula, J.E.; Espindola, L.S. In vitro antiplasmodial activity of Brazilian Cerrado plants used as traditional remedies. *Journal of Ethnopharmacology* **2007**, *110*, 165–170, doi:10.1016/j.jep.2006.09.015.
133. de Paula, R.C.; da Silva, S.M.; Faria, K.F.; Frézard, F.; Moreira, C.P. de S.; Foubert, K.; Lopes, J.C.D.; Campana, P.R.V.; Rocha, M.P.; Silva, A.F.; et al. In vitro antileishmanial activity of leaf and stem extracts of seven Brazilian plant species. *Journal of Ethnopharmacology* **2019**, *232*, 155–164, doi:10.1016/j.jep.2018.12.026.
134. da Silva, A.C.N.; do Nascimento, R.M.C.; Rodrigues, D.C. do N.; Ferreira, P.M.P.; Pessoa, C.; Lima, D.J.B.; Moraes Filho, M.O. de; de Almeida, R.M.; Ferreira, S.R.; Fujiwara, R.T.; et al. In vitro activity evaluation of seven Brazilian Asteraceae against cancer cells and *Leishmania amazonensis*. *South African Journal of Botany* **2019**, *121*, 267–273, doi:10.1016/j.sajb.2018.11.008.
135. Ribeiro, T.G.; Chávez-Fumagalli, M.A.; Valadares, D.G.; Franca, J.R.; Lage, P.S.; Duarte, M.C.; Andrade, P.H.R.; Martins, V.T.; Costa, L.E.; Arruda, A.L.A.; et al. Antileishmanial activity and cytotoxicity of Brazilian plants. *Experimental Parasitology* **2014**, *143*, 60–68, doi:10.1016/j.exppara.2014.05.004.
136. Alves, D.R.; Maia De Morais, S.; Tomiotto-Pellissier, F.; Miranda-Sapla, M.M.; Vasconcelos, F.R.; Silva, I.N.G. Da; Araujo De Sousa, H.; Assolini, J.P.; Conchon-Costa, I.; Pavanelli, W.R.; et al. Flavonoid Composition and Biological Activities of Ethanol Extracts of *Caryocar coriaceum* Wittm., a Native Plant from Caatinga Biome. *Evidence-based Complementary and Alternative Medicine* **2017**, *2017*, doi:10.1155/2017/6834218.
137. Tomiotto-Pellissier, F.; Alves, D.R.; Miranda-Sapla, M.M.; de Morais, S.M.; Assolini, J.P.; da Silva Bortoleti, B.T.; Gonçalves, M.D.; Cataneo, A.H.D.; Kian, D.; Madeira, T.B.; et al. *Caryocar coriaceum* extracts exert leishmanicidal effect acting in promastigote forms by apoptosis-like mechanism and intracellular amastigotes by Nrf2/HO-1/ferritin dependent response and iron depletion: Leishmanicidal effect of *Caryocar coriaceum* leaf exracts. *Biomedicine and Pharmacotherapy* **2018**, *98*, 662–672, doi:10.1016/j.biopha.2017.12.083.
138. Charneau, S.; de Mesquita, M.L.; Bastos, I.M.D.; Santana, J.M.; de Paula, J.E.; Grellier, P.; Espindola, L.S. In vitro investigation of Brazilian Cerrado plant extract activity against *Plasmodium falciparum*, *Trypanosoma cruzi* and *T. brucei gambiense*. *Natural Product Research* **2015**, *30*, 1320–1326, doi:10.1080/14786419.2015.1055264.
139. Santana, D.B.; da Costa, R.C.; Araújo, R.M.; de Paula, J.E.; Silveira, E.R.; Braz-Filho, R.; Espindola, L.S. Activity of Fabaceae species extracts against fungi and *Leishmania*: Vatacarpan as a novel potent anti-C*andida* agent. *Revista Brasileira de Farmacognosia* **2015**, *25*, 401–406, doi:10.1016/j.bjp.2015.07.012.
140. Morais-Costa, F.; Bastos, G.A.; Soares, A.C.M.; Costa, E.G.L.; Vasconcelos, V.O.; Oliveira, N.J.F.; Braga, F.C.; Duarte, E.R.; Lima, W.S. In vitro and in vivo action of *Piptadenia viridiflora* (Kunth) Benth against *Haemonchus contortus* in sheep. *Veterinary Parasitology* **2016**, *223*, 43–49, doi:10.1016/j.vetpar.2016.04.002.
141. Ribeiro, T.G.; Nascimento, A.M.; Henriques, B.O.; Chávez-Fumagalli, M.A.; Franca, J.R.; Duarte, M.C.; Lage, P.S.; Andrade, P.H.R.; Lage, D.P.; Rodrigues, L.B.; et al. Antileishmanial activity of standardized fractions of *Stryphnodendron obovatum* (Barbatimão) extract and constituent compounds. *Journal of Ethnopharmacology* **2015**, *165*, 238–242, doi:10.1016/j.jep.2015.02.047.
142. Farias, K.S.; Kato, N.N.; Boaretto, A.G.; Weber, J.I.; Brust, F.R.; Alves, F.M.; Tasca, T.; Macedo, A.J.; Silva, D.B.; Carollo, C.A. *Nectandra* as a renewable source for (+)-α-bisabolol, an antibiofilm and anti-*Trichomonas vaginalis* compound. *Fitoterapia* **2019**, *136*, 104179, doi:10.1016/j.fitote.2019.104179.
143. Calixto Júnior, J.T.; de Morais, S.M.; Gomez, C.V.; Molas, C.C.; Rolon, M.; Boligon, A.A.; Athayde, M.L.; de Morais Oliveira, C.D.; Tintino, S.R.; Henrique Douglas, M.C. Phenolic composition and antiparasitic activity of plants from the Brazilian Northeast "Cerrado." *Saudi Journal of Biological Sciences* **2016**, *23*, 434–440, doi:10.1016/j.sjbs.2015.10.009.
144. Cunha, W.R.; Dos Santos, F.M.; De Andrade Peixoto, J.; Veneziani, R.C.S.; Crotti, A.E.M.; Silva, M.L.A.; Da Silva Filho, A.A.; Albuquerque, S.; Turatti, I.C.C.; Bastos, J.K. Screening of plant extracts from the Brazilian Cerrado for their in vitro trypanocidal activity. *Pharmaceutical Biology* **2009**, *47*, 744–749, doi:10.1080/13880200902951361.
145. Charneau, S.; de Mesquita, M.L.; Bastos, I.M.D.; Santana, J.M.; de Paula, J.E.; Grellier, P.; Espindola, L.S. In vitro investigation of Brazilian Cerrado plant extract activity against *Plasmodium falciparum*, *Trypanosoma cruzi* and *T. brucei gambiense*. *Natural Product Research* **2015**, *30*, 1320–1326, doi:10.1080/14786419.2015.1055264.
146. Bou, D.D.; Tempone, A.G.; Pinto, É.G.; Lago, J.H.G.; Sartorelli, P. Antiparasitic activity and effect of casearins isolated from *Casearia sylvestris* on *Leishmania* and *Trypanosoma cruzi* plasma membrane. *Phytomedicine* **2014**, *21*, 676–681, doi:10.1016/j.phymed.2014.01.004.

147. Passos, A.; Rodríguez, R.; Ferreira, C.; Soares, C.; Somner, V.; Hamerski, L.; da Cunha Pinto, A.; Rezende, M.; Saraiva, E.M. Anti-*Leishmania amazonensis* activity of *Serjania lethalis* A. St.-Hil. *Parasitology International* **2017**, *66*, 940–947, doi:10.1016/j.parint.2016.10.018.
148. Moreira, R.R.D.; Martins, G.Z.; Magalhães, N.O.; Almeida, A.E.; Pietro, R.C.L.R.; Silva, F.A.J.; Cicarelli, R.M.B. In vitro trypanocidal activity of solamargine and extracts from *Solanum palinacanthum* and *Solanum lycocarpum* of brazilian cerrado. *Anais da Academia Brasileira de Ciencias* **2013**, *85*, 903–907, doi:10.1590/S0001-37652013000300006.
149. Clementino, L. da C.; Velásquez, A.M.A.; Passalacqua, T.G.; de Almeida, L.; Graminha, M.A.S.; Martins, G.Z.; Salgueiro, L.; Cavaleiro, C.; Sousa, M. do C.; Moreira, R.R.D. In vitro activities of glycoalkaloids from the *Solanum lycocarpum* against *Leishmania infantum*. *Revista Brasileira de Farmacognosia* **2018**, *28*, 673–677, doi:10.1016/j.bjp.2018.07.008.
150. Cordeiro, T.D.M.; Borghetti, F.; Caldas Oliveira, S.C.; Bastos, I.M.D.; De Santana, J.M.; Grellier, P.; Charneau, S. Brazilian cerrado *Qualea grandiflora* Mart. leaves exhibit antiplasmodial and trypanocidal activities in vitro. *Pharmacognosy Magazine* **2017**, *13*, 668–672, doi:10.4103/pm.pm_100_17.
151. Gonçalves Neto, G.; da Costa, R.; Zanini, C.; Aguiar, A.C.; de Souza, J.; de Souza, G.; Severino, R.; Cass, Q.; Cruz, F.; Oliva, G.; et al. Chemical Prospection of *Qualea grandiflora* Mart. Fruit and Stem Extracts and Their in vitro and in vivo Antiplasmodial Activity. *Journal of the Brazilian Chemical Society* **2020**, doi:10.21577/0103-5053.20200034.
152. De Mendonça, F.A.C.; Da Silva, K.F.S.; Dos Santos, K.K.; Ribeiro Júnior, K.A.L.; Sant'Ana, A.E.G. Activities of some Brazilian plants against larvae of the mosquito *Aedes aegypti*. *Fitoterapia* **2005**, *76*, 629–636, doi:10.1016/j.fitote.2005.06.013.
153. Rodrigues, A.M.S.; De Paula, J.E.; Degallier, N.; Molez, J.F.; Espíndola, L.S. Larvicidal activity of some Cerrado plant extracts against *Aedes aegypti*. *Journal of the American Mosquito Control Association* **2006**, *22*, 314–317, doi:10.2987/8756-971X(2006)22[314:LAOSCP]2.0.CO;2.
154. Massarolli, A.; Pereira, M.J.B.; Foerster, L.A. *Annona crassiflora* mart. (Annonaceae): Effect of crude extract of seeds on larvae of soybean looper *Chrysodeixis includens* (Lepidoptera: Noctuidae). *Bragantia* **2017**, *76*, 398–405, doi:10.1590/1678-4499.374.
155. Souza, C.M. de; Baldin, E.L.L.; Ribeiro, L. do P.; Santos, T.L.B. dos; Silva, I.F. da; Morando, R.; Vendramim, J.D. Antifeedant and growth inhibitory effects of Annonaceae derivatives on *Helicoverpa armigera* (Hübner). *Crop Protection* **2019**, *121*, 45–50, doi:10.1016/j.cropro.2019.03.008.
156. Ribeiro, L.P.; Domingues, V.C.; Gonçalves, G.L.P.; Fernandes, J.B.; Glória, E.M.; Vendramim, J.D. Essential oil from *Duguetia lanceolata* St.-Hil. (Annonaceae): Suppression of spoilers of stored-grain. *Food Bioscience* **2020**, *36*, 100653, doi:10.1016/j.fbio.2020.100653.
157. Peres, M.C.; de Souza Costa, G.C.; dos Reis, L.E.L.; da Silva, L.D.; Peixoto, M.F.; Alves, C.C.F.; Forim, M.R.; Quintela, E.D.; Araújo, W.L.; de Melo Cazal, C. In natura and nanoencapsulated essential oils from *Xylopia aromatica* reduce oviposition of *Bemisia tabaci* in *Phaseolus vulgaris*. *Journal of Pest Science* **2020**, *93*, 807–821, doi:10.1007/s10340-019-01186-6.
158. Silva, E.M.; Valencia, A.; Grossi-de-Sá, M.F.; Rocha, T.L.; Freire, É.; de Paula, J.E.; Espindola, L.S. Inhibitory action of Cerrado plants against mammalian and insect α-amylases. *Pesticide Biochemistry and Physiology* **2009**, *95*, 141–146, doi:10.1016/j.pestbp.2009.08.003.
159. Tavares, W.S.; Grael, C.F.F.; Menezes, C.W.G.; Cruz, I.; Serrão, J.E.; Zanuncio, J.C. Residual effect of extracts of native plants from brazil and a synthetic insecticide, chlorpyrifos, on *Coleomegilla maculata*, *Cycloneda sanguinea*, and *Eriopis connexa* (coleoptera: Coccinellidae). *Vie et Milieu* **2012**, *62*, 115–120.
160. DE MENEZES, C.W.G.; DE S. TAVARES, W.; DE SOUZA, E.G.; SOARES, M.A.; SERRÃO, J.E.; ZANUNCIO, J.C. Effects of crude extract fractions of *Adenocalymma nodosum* (Bignoniaceae) on duration of pupa stage emergence of *Tenebrio molitor* (Coleoptera: Tenebrionidae) and phytotoxicity on vegetable crops. *Allelopathy Journal* **2014**, *33*, 141–149.
161. Garcez, W.S.; Garcez, F.R.; da Silva, L.M.G.E.; Hamerski, L. Larvicidal activity against *Aedes aegypti* of some plants native to the West-Central region of Brazil. *Bioresource Technology* **2009**, *100*, 6647–6650, doi:10.1016/j.biortech.2009.06.092.
162. Santana, A.L.B.D.; Maranhão, C.A.; Santos, J.C.; Cunha, F.M.; Conceição, G.M.; Bieber, L.W.; Nascimento, M.S. Antitermitic activity of extractives from three Brazilian hardwoods against *Nasutitermes corniger*. *International Biodeterioration and Biodegradation* **2010**, *64*, 7–12, doi:10.1016/j.ibiod.2009.07.009.
163. Bezerra-Silva, P.C.; Santos, J.C.; Santos, G.K.N.; Dutra, K.A.; Santana, A.L.B.D.; Maranhão, C.A.; Nascimento, M.S.; Navarro, D.M.A.F.; Bieber, L.W. Extract of *Bowdichia virgilioides* and maackiain as larvicidal agent against *Aedes aegypti* mosquito. *Experimental Parasitology* **2015**, *153*, 160–164, doi:10.1016/j.exppara.2015.03.018.
164. Trindade, F.T.T.; Stabeli, R.G.; Pereira, A.A.; Facundo, V.A.; Silva, A. de A. *Copaifera multijuga* ethanolic extracts, oil-resin, and its derivatives display larvicidal activity against *Anopheles darlingi* and *Aedes aegypti* (Diptera: Culicidae). *Brazilian Journal of Pharmacognosy* **2013**, *23*, 464–470, doi:10.1590/S0102-695X2013005000038.
165. Alves, D.S. Toxicity of copaiba extracts to armyworm (*Spodoptera frugiperda*). *African Journal of Biotechnology* **2012**, *11*, 6578–6591, doi:10.5897/ajb11.196.
166. Rodrigues Macedo, M.L.; Freire, M.D.G.M.; Cabrini, E.C.; Toyama, M.H.; Novello, J.C.; Marangoni, S. A trypsin inhibitor from *Peltophorum dubium* seeds active against pest proteases and its effect on the survival of *Anagasta kuehniella* (Lepidoptera: Pyralidae). *Biochimica et Biophysica Acta - General Subjects* **2003**, *1621*, 170–182, doi:10.1016/S0304-4165(03)00055-2.
167. Oliveira, A.E.M.F.M.; Bezerra, D.C.; Duarte, J.L.; Cruz, R.A.S.; Souto, R.N.P.; Ferreira, R.M.A.; Nogueira, J.; da Conceição,

E.C.; Leitão, S.; Bizzo, H.R.; et al. Essential oil from *Pterodon emarginatus* as a promising natural raw material for larvicidal nanoemulsions against a tropical disease vector. *Sustainable Chemistry and Pharmacy* **2017**, *6*, 1–9, doi:10.1016/j.scp.2017.06.001.
168. Barbosa, C. da S.; Borges, L.M.F.; Nicácio, J.; Alves, R.D.; Miguita, C.H.; Violante, I.M.P.; Hamerski, L.; Garcez, W.S.; Garcez, F.R. In vitro activities of plant extracts from the Brazilian Cerrado and Pantanal against *Rhipicephalus (Boophilus) microplus* (Acari: Ixodidae). *Experimental and Applied Acarology* **2013**, *60*, 421–430, doi:10.1007/s10493-013-9656-z.
169. da SILVA, L.S.; Porfiro, C.A.; Silva, F.G.; Rodrigues, A.R.D.S.; Pereira, P.S. Acetylcholinesterase and α-amylase inhibitors from *Mouriri elliptica* Martius leaf extract. *Bioscience Journal* **2020**, *36*, 578–590, doi:10.14393/BJ-v36n2a2020-42714.
170. Magrini, F.E.; Specht, A.; Gaio, J.; Girelli, C.P.; Migues, I.; Heinzen, H.; Saldaña, J.; Sartori, V.C.; Cesio, V. Antifeedant activity and effects of fruits and seeds extracts of *Cabralea canjerana* (Vell.) Mart. (Meliaceae) on the immature stages of the fall armyworm *Spodoptera frugiperda* (JE Smith) (Lepidoptera: Noctuidae). *Industrial Crops and Products* **2015**, *65*, 150–158, doi:10.1016/j.indcrop.2014.11.032.
171. Coelho, A.A.M.; De Paula, J.E.; Espíndola, L.S. Insecticidal activity of cerrado plant extracts on *Rhodnius milesi* Carcavallo, Rocha, Galvão & Jurberg (Hemiptera: Reduviidae), under laboratory conditions. *Neotropical Entomology* **2006**, *35*, 133–138, doi:10.1590/S1519-566X2006000100018.
172. Sarmento, U.C.; Miguita, C.H.; de Oliveira Almeida, L.H.; Gaban, C.R.G.; da Silva, L.M.G.E.; de Souza, A.S.; Garcez, W.S.; Garcez, F.R. Larvicidal efficacies of plants from Midwestern Brazil: Melianodiol from *Guarea kunthiana* as a potential biopesticide against *Aedes aegypti*. *Memorias do Instituto Oswaldo Cruz* **2016**, *111*, 469–474, doi:10.1590/0074-02760160134.
173. Miguita, C.H.; Silva Da Barbosa, C.; Hamerski, L.; Sarmento, U.C.; Do Nascimento, J.N.; Garcez, W.S.; Garcez, F.R. 3β-O-tigloylmelianol from *Guarea kunthiana*: A new potential agent to control *Rhipicephalus (Boophilus) microplus*, a cattle tick of veterinary significance. *Molecules* **2015**, *20*, 111–126, doi:10.3390/molecules20010111.
174. Luiz, A.L.; Perlatti, B.; Marques, F.A.; Rodrigues-Filho, E.; Costa, E.N.; Ribeiro, Z.A.; Eduardo, W.I.; Boiça-Júnior, A.L.; Imatomi, M.; Gorecki, T.; et al. Efficacy of botanical extracts from Brazilian savannah against *Diabrotica speciosa* and associated bacteria. *Ecological Research* **2017**, *32*, 435–444, doi:10.1007/s11284-017-1454-3.
175. Oliveira, G.L.; Moreira, D. de L.; Mendes, A.D.R.; Guimarães, E.F.; Figueiredo, L.S.; Kaplan, M.A.C.; Martins, E.R. Growth study and essential oil analysis of *Piper aduncum* from two sites of Cerrado biome of Minas Gerais State, Brazil. *Brazilian Journal of Pharmacognosy* **2013**, *23*, 743–753, doi:10.1590/S0102-695X2013000500005.
176. De Souza Tavares, W.; Grazziotti, G.H.; De Souza, A.A.; De Sousa Freitas, S.; Consolaro, H.N.; De Aquino Ribeiro, P.E.; Zanuncio, J.C. Screening of extracts of leaves and stems of *Psychotria* spp. (Rubiaceae) against *Sitophilus zeamais* (Coleoptera: Curculionidae) and *Spodoptera frugiperda* (lepidoptera: Noctuidae) for maize protection. *Journal of Food Protection* **2013**, *76*, 1892–1901, doi:10.4315/0362-028X.JFP-13-123.
177. TavareS, W.D.S.; Faroni, L. riTa D.; Ribeiro, raFaeL C.; FouaD, hany ahmeD; FreiTaS, S.D.S.; Zanuncio, J.C. SEFFECTS OF ASTILBIN FROM *DIMORPHANDRA MOLLIS* (FABACEAE) FLOWERS AND BRAZILIAN PLANT EXTRACTS ON *SITOPHILUS ZEAMAIS* (COLEOPTERA: CURCULIONIDAE). *Florida Entomologist* **2014**, *97*, 892–901.
178. Fouad, H.A.; Faroni, L.R.D.A.; Tavares, W. de S.; Ribeiro, R.C.; Freitas, S. de S.; Zanuncio, J.C. Botanical extracts of plants from the Brazilian Cerrado for the integrated management of *Sitotroga cerealella* (Lepidoptera: Gelechiidae) in stored grain. *Journal of Stored Products Research* **2014**, *57*, 6–11, doi:10.1016/j.jspr.2014.01.001.
179. Costa, E.C.C.; Christofoli, M.; Costa, G.C. de S.; Peixoto, M.F.; Fernandes, J.B.; Forim, M.R.; Pereira, K. de C.; Silva, F.G.; Cazal, C. de M. Essential oil repellent action of plants of the genus *Zanthoxylum* against *Bemisia tabaci* biotype B (Homoptera: Aleyrodidae). *Scientia Horticulturae* **2017**, *226*, 327–332, doi:10.1016/j.scienta.2017.08.041.
180. Avelar Amado, P.; Fonsêca Castro, A.H.; Nunes Alves, S.; Brentan Silva, D.; Alexandre Carollo, C.; Alves Rodrigues dos Santos Lima, L. Phenolic compounds: antioxidant and larvicidal potential of *Smilax brasiliensis* Sprengel leaves. *Natural Product Research* **2020**, *34*, 2545–2553, doi:10.1080/14786419.2018.1543678.
181. Bezerra, J.C.B.; Silva, I.A.; Ferreira, H.D.; Ferri, P.H.; Santos, S.C. Molluscicidal activity against *Biomphalaria glabrata* of Brazilian Cerrado medicinal plants. *Fitoterapia* **2002**, *73*, 428–430, doi:10.1016/S0367-326X(02)00121-1.
182. Abreu, L.S.; do Nascimento, Y.M.; do Espirito-Santo, R.F.; Meira, C.S.; Santos, I.P.; Brandão, R.B.; Souto, A.L.; Guedes, M.L.S.; Soares, M.B.P.; Villarreal, C.F.; et al. Phenylpropanoids from *Croton velutinus* with cytotoxic, trypanocidal and anti-inflammatory activities. *Fitoterapia* **2020**, *145*, doi:10.1016/j.fitote.2020.104632.
183. Espindola, L.S.; Dusi, R.G.; Demarque, D.P.; Braz-Filho, R.; Yan, P.; Bokesch, H.R.; Gustafson, K.R.; Beutler, J.A. Cytotoxic triterpenes from *Salacia crassifolia* and metabolite profiling of Celastraceae species. *Molecules* **2018**, *23*, doi:10.3390/molecules23061494.
184. Nascimento, Y.M.; Abreu, L.S.; Lima, R.L.; Silva, A.D.S.; Costa, V.C.O.; Melo, J.I.M.; Scotti, M.T.; Sobral, M. V.; Araujo, S.S.; Filho, M.A.G.; et al. Zornioside, a dihydrochalcone C-glycoside, and other compounds from *Zornia brasiliensis*. *Revista Brasileira de Farmacognosia* **2018**, *28*, 192–197, doi:10.1016/j.bjp.2018.02.003.
185. Soares, P.R.O.; De Oliveira, P.L.; De Oliveira, C.M.A.; Kato, L.; Guillo, L.A. In Vitro antiproliferative effects of the indole alkaloid vallesiachotamine on human melanoma cells. *Archives of Pharmacal Research* **2012**, *35*, 565–571, doi:10.1007/s12272-012-0320-7.
186. Guterres, Z.R.; Garcez, F.R.; Garcez, W.S.; Silva, L.M.G.E.; Silva, A.F.G.; Duarte, C.U.N.B.D.; Batista-Silva, V.F. Evaluation of

the genotoxic activity of ethanol extract and secondary metabolites isolated from *Aiouea trinervis* Meisn. (Lauraceae). *Genetics and Molecular Research* **2014**, *13*, 972–979, doi:10.4238/2014.February.19.8.
187. Koolen, H.H.F.; Soares, E.R.; Da Silva, F.M.A.; De Oliveira, A.A.; De Souza, A.Q.L.; De Medeiros, L.S.; Rodrigues-Filho, E.; Cavalcanti, B.C.; Pessoa, C.O.; Moraes, M.O.; et al. Mauritic acid: A new dammarane triterpene from the roots of *Mauritia flexuosa* L.f. (Arecaceae). *Natural Product Research* **2013**, *27*, 2118–2125, doi:10.1080/14786419.2013.793685.
188. Mendonça Pauletti, P.; Araújo, A.R.; Young, M.C.M.; Giesbrecht, A.M.; Da Silva Bolzani, V. nor-Lignans from the leaves of *Styrax ferrugineus* (Styracaceae) with antibacterial and antifungal activity. *Phytochemistry* **2000**, *55*, 597–601, doi:10.1016/S0031-9422(00)00225-9.
189. de Sousa Andrade, L.M.; de Oliveira, A.B.M.; Leal, A.L.A.B.; de Alcântara Oliveira, F.A.; Portela, A.L.; de Sousa Lima Neto, J.; de Siqueira-Júnior, J.P.; Kaatz, G.W.; da Rocha, C.Q.; Barreto, H.M. Antimicrobial activity and inhibition of the NorA efflux pump of *Staphylococcus aureus* by extract and isolated compounds from *Arrabidaea brachypoda*. *Microbial Pathogenesis* **2020**, *140*, 103935, doi:10.1016/j.micpath.2019.103935.
190. Pereira, A.M.S.; Hernandes, C.; Pereira, S.I.V.; Bertoni, B.W.; França, S.C.; Pereira, P.S.; Taleb-Contini, S.H. Evaluation of anticandidal and antioxidant activities of phenolic compounds from *Pyrostegia venusta* (Ker Gawl.) Miers. *Chemico-Biological Interactions* **2014**, *224*, 136–141, doi:10.1016/j.cbi.2014.10.023.
191. Funari, C.S.; Gullo, F.P.; Napolitano, A.; Carneiro, R.L.; Mendes-Giannini, M.J.S.; Fusco-Almeida, A.M.; Piacente, S.; Pizza, C.; Silva, D.H.S. Chemical and antifungal investigations of six *Lippia* species (Verbenaceae) from Brazil. *Food Chemistry* **2012**, *135*, 2086–2094, doi:10.1016/j.foodchem.2012.06.077.
192. Gachet, M.S.; Kunert, O.; Kaiser, M.; Brun, R.; Zehl, M.; Keller, W.; Muñoz, R.A.; Bauer, R.; Schuehly, W. Antiparasitic compounds from *Cupania cinerea* with activities against *Plasmodium falciparum* and *Trypanosoma brucei rhodesiense*. *Journal of Natural Products* **2011**, *74*, 559–566, doi:10.1021/np100415m.
193. Lião, L.M.; Silva, G.A.; Monteiro, M.R.; Albuquerque, S. Trypanocidal activity of quinonemethide triterpenoids from *Cheiloclinium cognatum* (Hippocrateaceae). *Zeitschrift fur Naturforschung - Section C Journal of Biosciences* **2008**, *63*, 207–210, doi:10.1515/znc-2008-3-408.
194. Clementino, L. da C.; Velásquez, A.M.A.; Passalacqua, T.G.; de Almeida, L.; Graminha, M.A.S.; Martins, G.Z.; Salgueiro, L.; Cavaleiro, C.; Sousa, M. do C.; Moreira, R.R.D. In vitro activities of glycoalkaloids from the *Solanum lycocarpum* against *Leishmania infantum*. *Revista Brasileira de Farmacognosia* **2018**, *28*, 673–677, doi:10.1016/j.bjp.2018.07.008.
195. Nogueira, M.S.; Da Costa, F.B.; Brun, R.; Kaiser, M.; Schmidt, T.J. ent-pimarane and ent-kaurane diterpenes from *Aldama discolor* (Asteraceae) and their antiprotozoal activity. *Molecules* **2016**, *21*, doi:10.3390/molecules21091237.
196. Sarmento, U.C.; Miguita, C.H.; de Oliveira Almeida, L.H.; Gaban, C.R.G.; da Silva, L.M.G.E.; de Souza, A.S.; Garcez, W.S.; Garcez, F.R. Larvicidal efficacies of plants from Midwestern Brazil: Melianodiol from *Guarea kunthiana* as a potential biopesticide against *Aedes aegypti*. *Memorias do Instituto Oswaldo Cruz* **2016**, *111*, 469–474, doi:10.1590/0074-02760160134.
197. Fouad, H.A.; Faroni, L.R.D.A.; Tavares, W. de S.; Ribeiro, R.C.; Freitas, S. de S.; Zanuncio, J.C. Botanical extracts of plants from the Brazilian Cerrado for the integrated management of *Sitotroga cerealella* (Lepidoptera: Gelechiidae) in stored grain. *Journal of Stored Products Research* **2014**, *57*, 6–11, doi:10.1016/j.jspr.2014.01.001.
198. Batista Pereira, L.G.; Petacci, F.; Fernandes, J.B.; Corrêa, A.G.; Vieira, P.C.; Da Silva, M.F.G.F.; Malaspina, O. Biological activity of astilbin from *Dimorphandra mollis* against *Anticarsia gemmatalis* and *Spodoptera frugiperda*. *Pest Management Science* **2002**, *58*, 503–507, doi:10.1002/ps.478.
199. Cintra, P.; Bueno, F.C.; Bueno, O.C.; Malaspina, O.; Petacci, F.; Fernandes, J.B. Astilbin toxicity to leaf-cutting ant *Atta sexdens rubropilosa* (Hymenoptera: Formicidae). *Sociobiology* **2005**, *45*, 347–353.
200. Silva, R.L.; Demarque, D.P.; Dusi, R.G.; Sousa, J.P.B.; Albernaz, L.C.; Espindola, L.S. Residual Larvicidal Activity of Quinones against *Aedes aegypti*. *Molecules* **2020**, *25*, doi:10.3390/molecules25173978.
201. Napoleão, T.H.; Pontual, E.V.; De Albuquerque Lima, T.; De Lima Santos, N.D.; Sá, R.A.; Coelho, L.C.B.B.; Do Amaral Ferraz Navarro, D.M.; Paiva, P.M.G. Effect of *Myracrodruon urundeuva* leaf lectin on survival and digestive enzymes of *Aedes aegypti* larvae. *Parasitology Research* **2012**, *110*, 609–616, doi:10.1007/s00436-011-2529-7.
